# Serum metabolomics-driven network pharmacology elucidate the anti-rheumatoid arthritis potential of garden cress

**DOI:** 10.1038/s41598-025-13412-6

**Published:** 2025-09-01

**Authors:** Sarah A. Elsayed, Reham S. Ibrahim, El Moataz Bellah El Naggar, Yasmine A. Hassan, Eman Shawky

**Affiliations:** 1 Department of Pharmacognosy, Faculty of Pharmacy, Damanhur University, Damanhur, Egypt; 2 Department of Pharmacognosy, Faculty of Pharmacy, Alexandria University, Alexandria, 21521 Egypt; 3 Department of Pharmacology, Faculty of Pharmacy, Damanhur University, Damanhur, Egypt

**Keywords:** Garden cress, Rheumatoid arthritis, Functional food, Serum pharmacochemistry, Network pharmacology, Plant sciences, Systems biology

## Abstract

Garden cress (*Lepidium sativum* L.) has been traditionally utilized for the treatment of various diseases and is increasingly consumed as a functional food and alternative medicine in many countries due to its therapeutic potential. Notably, *L. sativum* is a promising candidate for mitigating rheumatoid arthritis (RA). This study employed a serum pharmacochemistry approach combined with a network pharmacology strategy to identify the active components and elucidate the underlying mechanisms of *L. sativum* in RA management. An RA rat model was established using Complete Freund’s Adjuvant (CFA). Following *L. sativum* administration, bioactive serum components were identified and quantified as markers of its pharmacological activity. Twenty-six serum metabolites, including 11 prototype compounds and 15 derived metabolites, were identified as key bioactive constituents absorbed at significant concentrations, potentially mediating the anti-RA effects of *L. sativum*. Among these, fatty acids and their conjugated metabolites emerged as the most relevant. Through network pharmacology, potential target genes and associated pathways were predicted. KEGG pathway analysis highlighted critical RA-related pathways, including arachidonic acid metabolism, modulation of inflammatory regulators in TRP channels, linoleic acid metabolism, and antifolate resistance pathways. Experimental data demonstrated that *L. sativum* significantly downregulated key inflammatory mediators such as IL-1β, TNF-α, MMP-9, CYP1A2, PLA2G2A, and MAPK8. This integrated study provides insight into the molecular mechanisms and active constituents of *L. sativum*, serving as a foundational reference for its therapeutic application against RA.

**Supplementary Information:**

The online version contains supplementary material available at 10.1038/s41598-025-13412-6.

## Introduction

In recent years, medicinal plants have gained significant global recognition due to their substantial importance as both nutritional resources and reservoirs of bioactive compounds. These plants are rich in diverse chemical constituents, including proteins, carbohydrates, minerals, vitamins, and essential fatty acids^1^ as well as a wide array of phytochemicals such as alkaloids, flavonoids, volatile oils, and other secondary metabolites. These bioactive constituents exhibit multifaceted pharmacological actions by targeting diverse molecular pathways, thereby contributing to the treatment and management of various diseases^2^.

Garden cress (*Lepidium sativum*), commonly referred to as “Hab alrashad” and belonging to the Brassicaceae family, is a fast-growing, erect annual herb widely cultivated in Egypt and Western Asia. Its cultivation has also extended to North Africa and Europe^3^. Various parts of the plant, including the roots, seeds, and leaves, are utilized both as a nutritional resource and for medicinal purposes^4^. Nutritionally, it is a rich source of proteins, carbohydrates, fibers, and essential minerals such as calcium, phosphorus, potassium, and zinc^5^. Medicinally, it is valued for its diverse phytochemical profile, which includes tannins, alkaloids (notably dimeric imidazole alkaloids such as lepidines B, C, D, E, and F)^4^, flavonoids, glycosides, glucosinolates^4^, and phenols^6^.

Rheumatoid arthritis (RA) is a chronic, systemic autoimmune disorder primarily characterized by persistent inflammation of the joints. If inadequately managed, RA can progress to affect vital organs, including the eyes, kidneys, heart, lungs, digestive system, and nervous system^7^. The disease causes severe joint damage, potentially leading to deformities and bone erosion. Despite advancements in medical science, no definitive cure for RA exists. Current therapeutic approaches focus on mitigating inflammation, alleviating pain, and preventing disease progression. Pharmacological management of RA involves the use of synthetic drugs. First-line treatments include non-steroidal anti-inflammatory drugs (NSAIDs) like naproxen and glucocorticoids such as corticosteroids. Second-line options comprise disease-modifying antirheumatic drugs (DMARDs) such as methotrexate (MTX), while biologics like abatacept and anakinra are used for more advanced cases. Although these medications effectively alleviate symptoms and manage disease progression, they are often associated with significant side effects. For instance, NSAIDs can cause gastrointestinal (GI) complications such as ulcers and bleeding, while DMARDs may lead to liver toxicity, bone marrow suppression, and cirrhosis. Additionally, the high cost of biologic therapies poses a financial burden^8^.

*Lepidium sativum* was traditionally used for long times as an efficient remedy to alleviate symptoms of RA due to its richness of antioxidants and secondary metabolites as flavonoids, alkaloids and glucosinolates^9^. It has been used for having anti -inflammatory properties^10^ through relieving joint pain and swelling. *L. sativum* with different parts as seeds, roots and leaves was consumed by many different cultures in different forms to achieve this goal. Its therapeutic activity returns to modulate immune response and reduce oxidative stress which are important key factors in RA etiology.

Prior to conducting the present *in-vivo* study, multiple lines of objective, measurable evidence from previously published, peer-reviewed literature provided scientific support for the potential therapeutic effects of *L. sativum*. In vitro studies have documented its ability to inhibit key inflammatory mediators and oxidative stress. Ahmad et al. demonstrated that polysaccharides contained in *L. sativum* seed powder significantly inhibited TNF-α production in *Escherichia coli*-stimulated mice, diminishing the pro-inflammatory response in a dose-dependent manner^11^. Similarly, Tounsi et al. (2022) reported that *L. sativum* extract could prevent the production of superoxide anion in peritoneal neutrophils obtained from albino mice, increase nitric oxide and glutathione levels, and scavenge reactive oxygen species (ROS), suggesting its potential as an antioxidant^12^.

Additional in vitro studies have corroborated the anti-inflammatory and antioxidant potential of *L. sativum*. Kadam et al. (2021) demonstrated that its seed extract exhibited membrane stabilization and protein denaturation inhibition, confirming its ability to suppress auto-antigen production and protein damage^13^. Aydemir and Becerik linked its phenolic content to potent antioxidant activity, showing that these compounds chelate free iron and inhibit oxidative chain reactions. Al-Sheddi et al. (2022) further validated its cytoprotective effects by showing that *L. sativum* inhibited hydrogen peroxide (*H₂O₂*)-induced oxidative stress in human liver cells, improving cell viability and reducing lipid peroxidation^14^.

Beyond in vitro studies, *L. sativum* has demonstrated significant anti-inflammatory and analgesic effects in various animal models. Al-Yahya et al. observed that its ethanolic extract significantly reduced carrageenan-induced edema and exhibited mild inhibition of granuloma formation in rats, while also showing analgesic effects in the hot plate test^15^. Raval & Ravishankar further confirmed its analgesic activity in Charles Foster albino rats and Swiss albino mice, noting inhibition of neurogenic and inflammatory pain responses^16^. Furthermore, Attia et al. highlighted its anti-inflammatory and antioxidant properties in a diabetic rat model, where *L. sativum* improved pancreatic histology and reduced inflammatory infiltration^17^.

Given this robust prior evidence from in vitro and animal model studies, the present research was designed to further elucidate the pharmacological mechanisms of *L. sativum* using an integrative serum metabolomics-driven approach in a well-established RA rat model. The use of animal models was justified to translate mechanistic insights into *in-vivo* conditions, complementing previous in vitro findings and supporting future clinical investigations.

In this study, the phytochemical composition of *L. sativum* was meticulously analyzed using UPLC-MS/MS, yielding a comprehensive chromatographic fingerprint that underscores its diverse array of bioactive constituents. Biological evaluations conducted on an *in-vivo* rheumatoid arthritis (RA) rat model demonstrated that administration of *L. sativum* at multiple dose levels significantly ameliorated RA symptoms. The pharmacological actions of natural products are widely attributed to their active components. However, the link between these components and the products' therapeutic benefits remains unclear for most natural products^18^. To overcome this challenge, the discipline of serum pharmacochemistry was introduced. This field assumes that once active ingredients are absorbed into the bloodstream, they can begin to act on the body. Serum pharmacochemistry employs the UPLC-MS/MS technique as a dependable and swift way to monitor these ingredients once they enter the bloodstream.

Despite abundant *in-vitro* and preliminary *in-vivo* evidence supporting *L. sativum*'s anti-inflammatory and antioxidant properties, a critical gap exists in the lack of prior studies that have systematically identified the *in-vivo* absorbed bioactive components or elucidated their molecular mechanisms against RA using integrative modern approaches. Traditional phytochemical studies have focused on crude extracts without linking the absorbed active metabolites to pharmacological targets in the context of RA. Moreover, the pathophysiology of RA is multifactorial, involving complex signaling cascades, necessitating a holistic, systems-level analysis.

Serum metabolomics-driven analysis enables the identification of compounds that reach systemic circulation at pharmacologically relevant concentrations, thereby providing a realistic basis for understanding the bioactive entities responsible for therapeutic effects. By combining this approach with network pharmacology, which systematically maps compound-target-pathway interactions, we can decode the multi-target and multi-pathway mechanisms underlying *L. sativum* anti-RA effects.

This integrated strategy overcomes the limitations of isolated compound studies by capturing the dynamic biological behavior of plant metabolites and correlating it with RA pathogenesis. Notably, to our knowledge, this study represents the first attempt to employ a serum pharmacochemistry-based network pharmacology approach to investigate *L. sativum* against rheumatoid arthritis, offering new mechanistic insights and paving the way for rational development of plant-based interventions for autoimmune diseases.

By integrating serum pharmacochemistry with network pharmacology, we identified key phytochemicals that interact with specific molecular targets implicated in RA pathophysiology. This approach emphasizes the critical role of multidisciplinary methodologies in advancing the understanding of disease mechanisms and optimizing therapeutic strategies.

## Materials and methods

### Chemicals and reagents

Complete Freund’s Adjuvant (CFA) (10 mg/ml of heat- killed and dried *Mycobacterium tuberculosis* in oil emulsion) is purchased from Sigma- Aldrich (St. Louis, MO, USA) stored at 2–8 °C., methotrexate (Hikma Pharmaceuticals Co., Egypt), ethyl alcohol 70% (International Company for Sup. &Med. Industries, Egypt), Methanol (SDFCL, India). All other chemicals and solvents used were of analytical grade.

### Preparation of *L. sativum* extract

*L. sativum* seeds were purchased from the market and authenticated by Professor Mohamed Abd El-Sattar, Department of Crop Science, Faculty of Agriculture, Alexandria University. First, seeds were ultrasonicated in ethyl alcohol 70% at 45 °C and 300 W for 30 min in an ultrasonic water bath, after grinding them smoothly and the supernatant was filtered twice. Then, the filtrate was evaporated to dryness using a rotary evaporator at 50–55 °C and vacuum pressure at 100 par tailed by a vacuum-supported freeze –drying apparatus to get extract in dry status and held in reserve at 2–8 °C for later use. Pharmacological studies were held on the dry extract after dissolving it in distilled water. The doses used are expressed as mg of dried extract per kg body weight.

### Experimental design

#### Animals

Male western albino rats (170 ± 20 g) were purchased from National Research Centre (Dokki, Giza, Egypt). Rats were housed in cages at 24 ± 1 °C, under a 12 h light–dark cycle, with ad libitum access to food and water. Rats were allowed to acclimatize to these conditions for a minimum of 7 days prior to the experiment. All animal experiments were conducted in accordance with the guidelines approved by the Ethics Committee of Faculty of Pharmacy, Damanhur University (approval no. 323PG8). The study is reported in accordance with ARRIVE guidelines (https://arriveguidelines.org).

#### Induction of RA rat, experimental setup and anti-RA effect evaluation

Arthritis was induced by subcutaneous injection of a single dose (0.3 ml) of CFA (1 mg/ml) into the plantar surface of the left hind paw, as described previously^19^. Seven days after CFA injection, RA rats were randomly divided into five groups (n = 6 per group) and were intraperitoneal (i.p.) administered one of the following: Saline, 0.75 mg/kg of methotrexate (MTX)^20^, 100 mg/kg of *L. sativum* " low dose group", 250 mg/kg of *L. sativum* "medium dose group", and 400 mg/kg of *L. sativum* "high dose group" for 20 d. Rats in the control group were injected with saline in the same manner as injection of CFA. Rat body weight, the diameter of the left hind paw and the arthritic score were recorded every three days after CFA injection (recorded as day 0). The arthritis score criteria were based on a 0–4 scale as follows: negative erythema or swelling "-" was recorded as 0; mild erythema or swelling "+ " was recorded as 1; moderate edema " ++ " was recorded as 2; joint function limitation "+++" was recorded as 3; excessive joint edema and joint stiffness "++++ " was recorded as 4^21^. At day 27, rats were anesthetized using thiopental (50 mg/kg, i.p.), rat blood was collected by cardiac puncture and the serum rat blood was collected and the serum was separated by centrifugation at 3500 rpm and 4 °C for 15 min. Serum samples were frozen at –80 °C until later analysis. After blood collection, rats were rapidly euthanized with an overdose of thiopental (150 mg/kg, i.p.) and spleen and thymus were collected. The organ index was expressed as the ratio of organ wet weight to rat body weight (g/g): Organ index = organ wet weight (g)/animal weight (g) × 100%. Knee joints were harvested and fixed in buffered formalin-saline (10%) at room temperature for 48 h for histopathological examinations.

### Serum samples collection and preparation

Serum samples were prepared for serum pharmacochemistry analysis by protein precipitation with methanol. 3 ml equal amount of defrosted serum and 11 ml of chromatography grade methanol was uniformly mixed for 60 s, and centrifuged at 4000 rpm for 20 min to extricate particulates and proteins. Subsequently, the supernatant was filtrated through 0.22 μm microporous membrane and dried using a nitrogen stream at 40 ^◦^C. Methanol (100 μl) was instilled to dissolve the residue, with a 10 μl aliquot being used for chromatographic analysis.

### UPLC- MS/MS parameters and conditions

Raw LC–MS data were introduced to MZmine 2.0 (http: //mzmine. sourceforge.net/) for data attainment and management. The processing parameters were: mass range 100–1000 Da, RT tolerance (min) 0.1, signal-to-noise threshold 1.5. Hence, a data matrix containing the retention time, mass/charge ratio (m/z), and peak intensity was well-known. The crude components and metabolites in the *Lepidium*-dosed plasma were explained based on chromatographic elution means, a self-built database rationally characterized the components of *Lepidium*, mass fragment patterns (quasi-molecular ions as well as diagnostic multilevel ion fragments) and tracking the reference standards and related literature. For details of UPLC- MS/MS parameters and conditions, please refer to supplementary material.

### Network pharmacology analysis

Since the absorbed components of the extract have the effect on the targets^22^, therefore, the SMILES (Simplified Molecular-Input Line-Entry System) strings of those compounds were acquired from PubChem database (https://pubchem.ncbi.nlm.nih.gov/) and followed by introducing them to STITCH database (http://stitch.embl.de/), SwissTargetPrediction database (http://www.swisstargetprediction.ch/) with the species selected as "*Homo sapiens*" and similarity ensemble approach (SEA) (https://sea.bkslab.org/) whereas the genes involved in RA were gained from GeneCards (http://genecards.org/) by typing (Rheumatoid arthritis). Then, the drug components and genes related to RA were introduced to VENNY 2.1.0 (https://csbg.cnb.csic.es/BioinfoGP/venny.html). A drug-disease common genes list was assessed for the treatment of RA.

Significantly, the corresponding targets associated with these bioactive components were transformed into official gene symbols using the UniProtKB search function in UniProt (https://www.uniprot.org/).

The overlapping common genes were then submitted to STRING version 12.0 (https://string-db.org/), a comprehensive biological database capable of developing protein–protein interaction (PPI) networks. The species was set as "Homo sapiens," and protein interactions with a confidence interaction score greater than 0.6 were selected.

To further explore the biological functions and mechanisms of the identified targets, gene ontology (GO) and Kyoto Encyclopedia of Genes and Genomes (KEGG) pathway enrichment analyses (www.kegg.jp/kegg/kegg1.html) were performed using STRING. The list of common genes obtained from VENNY (https://bioinfogp.cnb.csic.es/tools/venny/index2.0.2.html) was introduced into STRING, again setting "*Homo sapiens*" as the organism.

Finally, pharmacological networks were constructed and analyzed using Cytoscape 3.10.1 software to systematically decrypt the key genes and signaling pathways targeted by Lepidium sativum in RA treatment. The Network Analyzer tool was utilized to evaluate the degree of involvement and significance of nodes in each constructed network.

### Assessment and endorsement of the anti-RA effect of *L. sativum* in rats

#### Biochemical assays of serum samples

##### Measurement of the serum levels of TNF-α, IL-1β, MMP-9, PLA2G2A, MAPK8 and CYP 1A2 using ELISA

TNF-α, IL-1β, MMP-9 biomarkers were selected to be measured being the mostly common makers of inflammation. So, PLA2G2A, MAPK8 and CYP 1A2 were measured based on being the most related genes incorporated in the top RA-related pathways represented as arachidonic acid metabolism, inflammatory mediator regulation of TRP channels, linoleic acid metabolism and drug metabolism-cytochrome P450 as inspired from (KEGG) pathway enrichment analysis. TNF-α, IL-1β, MMP-9, PLA2G2A, MAPK8 and CYP 1A2 levels were assessed in serum samples using ELISA, a technique that offers a reliable quantitative method with high sensitivity and specificity^23^. These markers were measured according to the manufacturer’s protocol. The values are presented as the mean ± SEM.

##### Measurement of serum liver enzymes levels

In order to assure the safety of *L. sativum* and to select the optimum dose of extract that doesn’t cause significant toxicity**,** ALT (Alanine aminotransferase) and AST (Aspartate aminotransferase) serum levels were evaluated using colorimetric assay with commercial kit according to manufacturer’s protocol^24^.

#### Histopathological analysis

Knee joints were decalcified in 10% EDTA, subsequently embedded in paraffin, sectioned into 5 μm thick slices, and stained with hematoxylin & eosin (H&E). Histopathological examination of rats′ joints was held at Nawah scientific center (Al-Asmarat, Almokattam, Cairo, Egypt). Joint destruction was scored as follows: 0: normal; 1: mild cartilage/bone erosion; 2: moderate erosion; 3: severe erosion with loss of normal joint architecture^25^.

### Statistical analysis

Statistical analysis was performed using GraphPad Prism 8 software package (GraphPad Software Inc., CA, USA). All data are expressed as mean ± standard error of mean (SEM), and the differences between experimental groups were assessed using one-way analysis of variance ANOVA followed by post hoc Tukey’s test. Kruskal–Wallis test followed by Dunnʾs multiple comparison test was used for analyzing arthritis score. P value < 0.05 was considered statistically significant.

## Results and discussion

### Identification of *L. sativum* extract compounds

The UPLC-MS/MS analysis identified and annotated 41 compounds in the *L. sativum* extract. A comprehensive list of these compounds, including their MS/MS fragmentation patterns and corresponding chemical classes, is provided in Table S1. The annotated phytoconstituents were primarily classified into amino acids, fatty acids, alkaloids, glucosinolates, carbohydrates, flavonoids, and phenolic glycosides. Additionally, the UPLC-MS/MS base peak chromatograms of the ethanolic extract of *L. sativum* in both negative and positive ionization modes are included as supplementary material (Fig. S1).

### Description of absorbed compounds in rat plasma after extract administration

UPLC-MS/MS analysis and serum pharmacochemistry approach were proceeded on the serum gathered after *in-vivo L. sativum* extract administration to RA rats to chemically outline and structurally distinguish nominee bioactive components using UPLC-MS/MS in positive and negative ion modes.

**Fig. 1:**
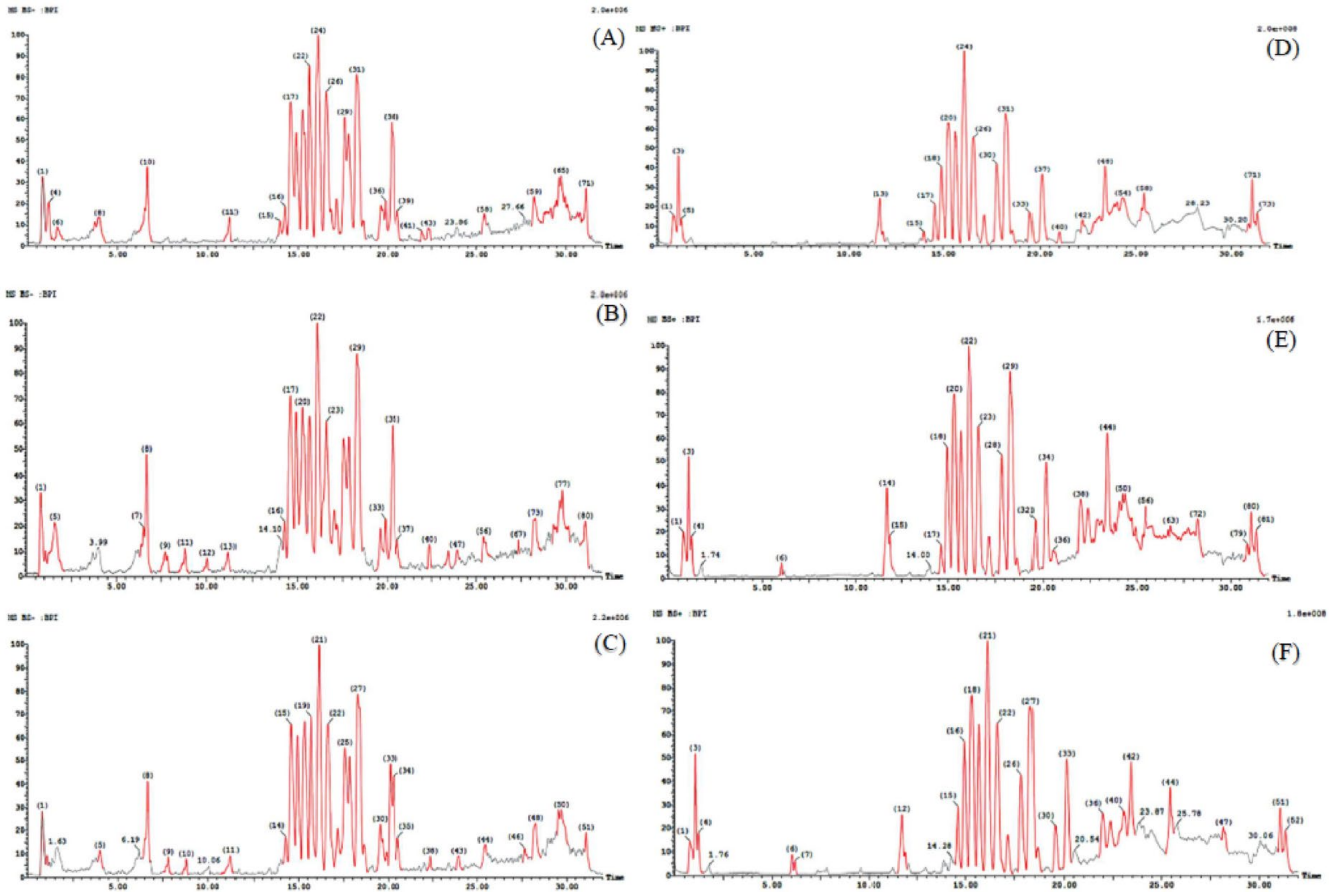
UPLC-MS/MS base peak chromatograms of plasma of RA-rats treated by *L*. *sativum* ethanolic extract in negative ionization mode. 100 mg/kg of *L. sativum* "low dose group" (**A**), 250 mg/kg of *L. sativum* "medium dose group" (**B**), 400 mg/kg of *L. sativum* "high dose group" (**C**) and in positive ionization mode. 100 mg/kg of *L. sativum* "low dose group" (**D**), 250 mg/kg of *L. sativum* "medium dose group" (**E**), 400 mg/kg of *L. sativum* "high dose group" (**F**) chromatograms.

Table 1 provides a comprehensive summary of the absorbable compounds extracted from dosed bio-samples of RA rats, analyzed using the UPLC-MS/MS technique. This includes detailed structural information such as retention time, mass measurements, distinguishing fragment ions, molecular formulas, and potential biotransformation pathways. Figure 1 presents the base peak chromatograms of plasma samples from RA rats treated with three dose levels of the extract. In this study, 26 compounds were identified, comprising 11 parent compounds and 15 metabolites in the serum samples. The absorbed compounds primarily belonged to phytochemical classes such as flavonoids, fatty acids, alkaloids, glucosinolates, carbohydrates, amino acids, and phenolic glycosides.

**Table 1:** Identification of absorbed components in rats’ plasma after oral administration of *L. sativum* extract by UPLC-MS/MS.

No	RT (min)	Identified compounds	Quasi molecular –ions	Measured mass(m/z)	Formula	MS/MS data	Transformations	References
M 1	0.79	Aspartic acid	[M-H]^-^	308.06	C_10_H_15_NO_10_	132, 88	O-Glucuronidation of aliphatic acids	^28^
1	0.81	Proline	[M + H]^+^	116.13	C_5_H_9_NO_2_	72	Prototype	^29^
2	3.26	Cysteine	[M + H]^+^	122.16	C_3_H_7_NO_2_S	77	Prototype	^30^
M 2	8.67	Benzyl isothiocyanate	[M + H]^+^	457.12	C_18_H_24_N_4_O_6_S_2_	150	GSH-Conjugation of isothiocyanate	^31^
3	9.97	Kaempferol-7-hexoside	[M + H]^+^	433.38	C_21_H_20_O_10_	271, 151, 133	Prototype	^32^
4	10.99	Glucobrassicin	[M-H]^-^	447.46	C_16_H_20_N_2_O_9_S_2_	367, 205	Prototype	^33^
M 3	14.81	Glucobrassicin	[M-H]^-^	285.00	C_10_H_10_N_2_O_4_S_2_	205	Glycoside hydrolysis	^34^
M 4	14.96	3,4,5-Trimethoxybenzyl glucosinolate	[M + H]^+^	515.11	C_18_H_28_NO_12_S_2_	482, 420, 258	Thioester S-methylation	^34^
M 5	15.05	Glucotropaolin	[M + H]^+^	490.01	C_14_H_19_NO_12_S_3_	327, 309, 224, 165	Sulfation of 1^ry^ & 2^ry^ alcohol	^4^
M6	15.55	Benzyl glucosinolate	[M + H]^+^	489.00	C_14_H_18_NO_12_S_3_	407, 276, 242	Sulfation of 1^ry^ alcohol	^35^
M7	16.01	Lepidine B/E/F	[M + H]^+^	523.19	C_26_H_27_N_4_O_8_	347, 174	N-Glucuronidation of azole	^4^,^28^
5	16.08	4-Methoxyglucobrassicin	[M + H]^+^	479.50	C_17_H_22_N_2_O_10_S_2_	399, 237	Prototype	^33^
M8	16.16	4-Methoxyglucobrassicin	[M-H]^-^	492.08	C_18_H_25_N_2_O_10_S_2_	316	Thioester S-methylation	^34^
6	16.61	Macathioamide A	[M-H]^-^	283.37	C_16_H_16_N_2_OS	240, 212, 168	Prototype	^36^
7	18.45	Lepidine B /E/F	[M + H]^+^	347.39	C_20_H_18_N_4_O_2_	174	Prototype	^4^
M9	18.45	Lepidine E	[M + H]^+^	331.15	C_20_H_18_N_4_O	158	4'-Dehydroxylation of substituted benzene	^4^
M10	19.87	Myristoleic acid	[M-H]^-^	281.20	C_16_H_29_NO_3_	225, 181	Glycine conjugation	^37^
M11	21.99	Lepidimoic acid	[M-H]^-^	319.06	C_12_H_16_O_10_	275	Dehydrogenation of 2^ry^ alcohol	^38^
8	22.37	Lepidine AK	[M + H]^+^	361.41	C_21_H_20_N_4_O_2_	188	Prototype	^4^
M12	22.37	9,11,14-Eicosatrienoic acid	[M-H]^-^	362.27	C_22_H_37_NO_3_	306	Glycine conjugation	^37^
M13	24.34	Nervonic acid	[M-H]^-^	337.31	C_22_H_42_O_2_	293	Beta oxidation of carboxylic acid	^38^
M14	28.21	Methyl isoheptadecanoate	[M-H]^-^	269.24	C_17_H_34_O_2_	73	Hydrolysis of carboxylic acid ester	^39^
9	30.04	Macaridine	[M + H]^+^	216.25	C_13_H_13_NO_2_	198, 169, 152	Prototype	^40^
M15	31.07	Alpha-linolenic acid	[M-H]^-^	293.21	C_18_H_30_O_3_	233, 69, 59	Aliphatic hydroxylation	^41^,^42^
10	31.10	Apetalumoside A	[M-H]^-^	801.67	C_34_H_42_O_22_	639, 477, 315	Prototype	^43^
11	31.20	Myristoleic acid	[M-H]^-^	225.34	C_14_H_26_O_2_	181	Prototype	^37^

Basically, the phase I metabolic pathways primarily included hydrolysis^26^, hydration, methylation, dehydration and hydroxylation^27^. Conversely, the main metabolic pathways of phase II integrate glucuronidation, glycine conjugation^27^ and GSH-Conjugation^26^.

#### Characterization of fatty acids

Fatty acids constitute a wide class of compounds and also rule the chemical composition of lepidium extract represented in saturated and unsaturated forms^44^. Previous researches proved that these compounds exerts an important effect in management of diverse inflammatory diseases^6^.

In the current analysis, we identified four fatty acids and one fatty acid ester at rat plasma after oral administration of lepidium extract. Among them, compound (**11)** was certainly identified as myristoleic acid by referring to literature data with a [M–H]^-^ at m/z 225.34 and an intense fragment at m/z 181 explained by loss of CO_2_^37^.

In our research, glycine conjugation is one of the metabolic pathways acquired by metabolites **(M10 and M12)**. Myristoleic acid metabolite **(M10)** exerting a predominant molecular ion peak of [M–H]^-^ at m/z 281.20 which was 56 Da higher than that of myristoleic acid, indicating that it was conjugated with glycine and undergone glycine conjugation phase ΙΙ reaction. Added to further fragmentation of a notable ion at m/z 181 corresponding to neutral loss of CO_2_ (44 Da). Meanwhile, **M12** was found to be 9,11,14-eicosatrienoic acid which exerts a predominant peak of [M–H]^-^ at m/z 362.27 and an intense fragment at m/z 306 which represents the loss of 56 Da indicating the presence of glycine moiety.

Furthermore, hydroxylation was also an important metabolic pathway for fatty acids where a hydroxylated product was identified as metabolite **(M15)** with a molecular ion peak of [M–H]^-^ at m/z 293.21 explained by hydroxylation of alpha-linolenic acid molecular ion peak (m/z 277) by (16 Da). Its MS/MS spectrum showed distinguished fragment ion at m/z 233 due to the loss of CO_2_ (44 Da) and other two fragment peaks at m/z 69 rational to (C_5_H_9_) and a fragment at m/z 59 (C_2_H_3_O_2_) due to the loss of [M–H–C_13_H_20_O_2_]—and [M–H–C_16_H_26_]^-^ , respectively^42^.

Hydrolysis of carboxylic acid ester is another metabolic pathway adopted by metabolite **(M14)** as a peak was identified at m/z 269.24 with a distinguishing Mclafferty rearrangement fragment at m/z 73 which submitted that it is a methyl ester of saturated fatty acid^45^. Thus, it was identified as methyl isoheptadecanoate.

#### Characterization of glucosinolates

Glucosinolates are a characteristic class to family Brassicaceae and specially *Lepidium* genus. they are decomposed at PH 5–7 and in presence of myrosinase to glycone and aglycone part which could be isothiocyanate, indoles, thiocyanates, nitriles or epithionitriles^34^. In our analysis, two prototypes (Glucobrassicin and 4-Methoxyglucobrassicin) and five metabolites of (Benzyl isothiocyanate, Glucobrassicin, 3,4,5-Trimethoxybenzyl glucosinolate, Glucotropaolin and 4-Methoxyglucobrassicin) were detected in rat serum treated with *Lepidium* extract. Peak **(M6)** was proposed as a sulfate conjugate and gave rise to a protonated ion of [M + H]^+^ at m/z 489.01 as a molecular ion peak. An intense fragment ion presents at m/z 407 [M + H–82]^+^ resulted from initial loss of the weakly bound sulfate group (82 Da) followed by characteristic signals at m/z 276 and 242 related to (C6H12O8S2) and (C6H10O8S) moieties, respectively. MS2 spectrum of benzyl glucosinolate further confirmed by previously reported data^35^.

Compounds **(4)** and **(5)** with a [M–H]^-^ at m/z 447.46 and [M + H]^+^ at m/z 479.50 were clearly characterized as glucobrassicin and 4-methoxyglucobrassicin, respectively by comparing with literature data^33^. Their MS2 spectra provided typical fragment ion peaks at m/z 367 and 399, respectively due to initial loss of SO_3_ (80 Da) and further fragment ion peaks at m/z 205 and 237, respectively due to loss of the glycone part (162 Da). A molecular ion peak of [M + H]^+^ appeared at m/z 285 corresponding to **(M3)** which evoked a fragment ion peak at m/z 205 resulted from hydrolysis of glucobrassicin and departure of SO_3_ (80 Da)^34^.

Methylation is a basic metabolic pathway as presented in **(M4 & M8)**. 3,4,5-trimethoxybenzyl glucosinolate **(M4)** is supposed to undergo S-methylation phase I reaction, exerting a predominant peak at m/z 515.11 with a mass shift of 15 Da consistent with methyl chain moiety (CH_3_) over the molecular ion peak of 3,4,5-trimethoxybenzyl glucosinolate (m/z 500). Then, its high-energy spectrum provided main product ions at m/z 482 due to a neutral loss of H2O by 18 Da, at m/z 420 corresponding to the loss of SO3 by (80 Da) and at m/z 258 related to the subsequent loss of glucose moiety^33^. GSH-Conjugation is another phase II metabolic pathway expected to be behave by **(M2)** as a precursor peak of [M + H]^+^ appeared at m/z 457.12 with a mass shift of (307 Da ) as a neutral loss of glutathione moiety^31^ exerting another peak of [M + H]^+^ at m/z 150 indicating the presence of benzyl isothiocyanate which is on the occasion of being a product of benzyl glucosinolate degradation^31^.

#### Characterization of alkaloids

*L.sativum* concludes different types of alkaloids as N-hydroxypyridine derivatives and imidazole alkaloids. In our study, three prototype compounds and two metabolites were detected in rat serum treated with *Lepidium* extract. Compound **(7)** showed a precursor peak of [M + H]^+^at m/z 347.39 which is characteristic to compounds lepidine B/E/F and undergo further fragmentation to define a predominant peak at m/z 174 coinciding with the loss of (C_10_H_9_N_2_O) moiety by (173 Da)^4^. Compound **(9)** with a molecular ion [M + H]^+^ at m/z 216.25 and fragment ion peaks at m/z 198 and m/z 169 due to the loss of a H_2_O molecule then HCO group, correspondingly. The extra neutral loss of the NH_3_ group from the later ion (m/z 169) produced the fragment at m/z 152. That proved the compound was macaridine.

N-Glucuronidation of azole is a phase II metabolic pathway which is adopted by metabolite **(M7)** as a precursor ion peak of [M + H]^+^ appeared at m/z 523.19 that was characterized as lepidine B/E/F glucuronide. Tandem mass of this peak provided a promising signal at m/z 347 evidently explained by glucuronide moiety (176 Da) loss from the parent ion which further fragmented yielding a product signal at m/z 174 after the loss of (C_10_H_9_N_2_O) moiety (173 Da) supporting the occurrence of lepidine B/E/F glucuronide^28^,^4^. Metabolite **(M9)** showed a precursor peak of [M + H]^+^ at m/z 331.15 indicating loss of (OH) moiety by ( 16 Da) of the parent compound (m/z 347) as it undergo 4'-dehydroxylation of substituted benzene and exerts a fragmentation ion peak at m/z 158 due to the loss of (C_10_H_9_N_2_O) moiety (173 Da) to identify the compound as lepidine B/E/F^46^. Similarly, compound (**8)** showed a [M + H]^+^ molecular ion peak at m/z 361.41 and a fragment peak at m/z 188 that reflects the loss of (C_10_H_9_N_2_O) moiety (173 Da) to identify the compound as lepidine AK^4^.

#### Characterization of amino acids

*L. sativum* extract is rich with high percentage of amino acids represented as essential and non-essential ones^47^ and that is reflected in rat serum where three of them were detected. Compounds (**1 & 2)** founded as prototypes while metabolite **(M1)** is the glucuronide form of aspartic acid. Related to compound **(1),** it has a precursor peak at m/z 116.1 and two fragment ion peak at m/z 72 due to neutral loss of CO_2_ by (44 Da). So, it could be identified as proline^41^.

Meanwhile, compound (**2)** exerted a precursor ion peak of [M + H]^+^ at m/z 122.16 and a product ion peak at m/z 77 by the loss of 45 Da indicating egression of (COOH) moiety. So, it was believed to be cysteine.

O-Glucuronidation of aliphatic acids is a phase II metabolic pathway which is adopted by metabolite **(M1)** as a precursor ion peak [M–H]^-^ appeared at m/z 308.06 that was characterized as aspartic acid glucuronide. Tandem mass of this peak provided a promising signal at m/z 132 evidently explained by glucuronide moiety (176 Da) loss from the parent ion which further fragmented yielding a product signal at m/z 88 after the loss of CO_2_ (44 Da) supporting the occurrence of aspartic acid^28^.

#### Characterization of miscellaneous compounds

Flavonoids is a wide class of secondary metabolites which constitute a relative percentage in *L. sativum* extract^47^. Our study recognized compound **(3)** which showed a molecular ion peak of [M + H]^+^ at m/z 433.38 and a fragment ion peak at m/z 271 which represents the kaempferol component^48^ which give means to the loss of sugar portion (C_6_H_10_O_5_) by 162 Da. Further fragmentation to kaempferol part to give two peaks at 151 and 133 as a result of Retro Diels–Alder reaction (RDA)^32^ and the compound was well identified as kaempferol-7-hexoside.

Related to compound **(6),** it is recognized as macathioamide A, as it produced a molecular ion peak of [M–H]^-^at m/z 283.37 and a fragment ion peak appeared at m/z 240 due the loss of (CONH) group, progressive fragmentation took place and a peak appeared at m/z 212 as a result of C = O loss by (28 Da) , followed by successive loss of C = S moiety by 44 Da and the peak appeared at m/z 168^36^.

Compound **(10)** is identified as apetalumoside A, showed a molecular ion peak of [M–H]^-^ at m/z 801.67 and a fragmentation ion peak at m/z 639 as a result of glucose moiety loss. Another two fragment peaks at m/z 477 and 315 due to (RDA) reaction^43^.

### Network pharmacology analysis

#### Scavenging of RA-associated target genes of *in-vivo* constituents of *L. sativum* and PPI network analysis

**Fig. 2:**
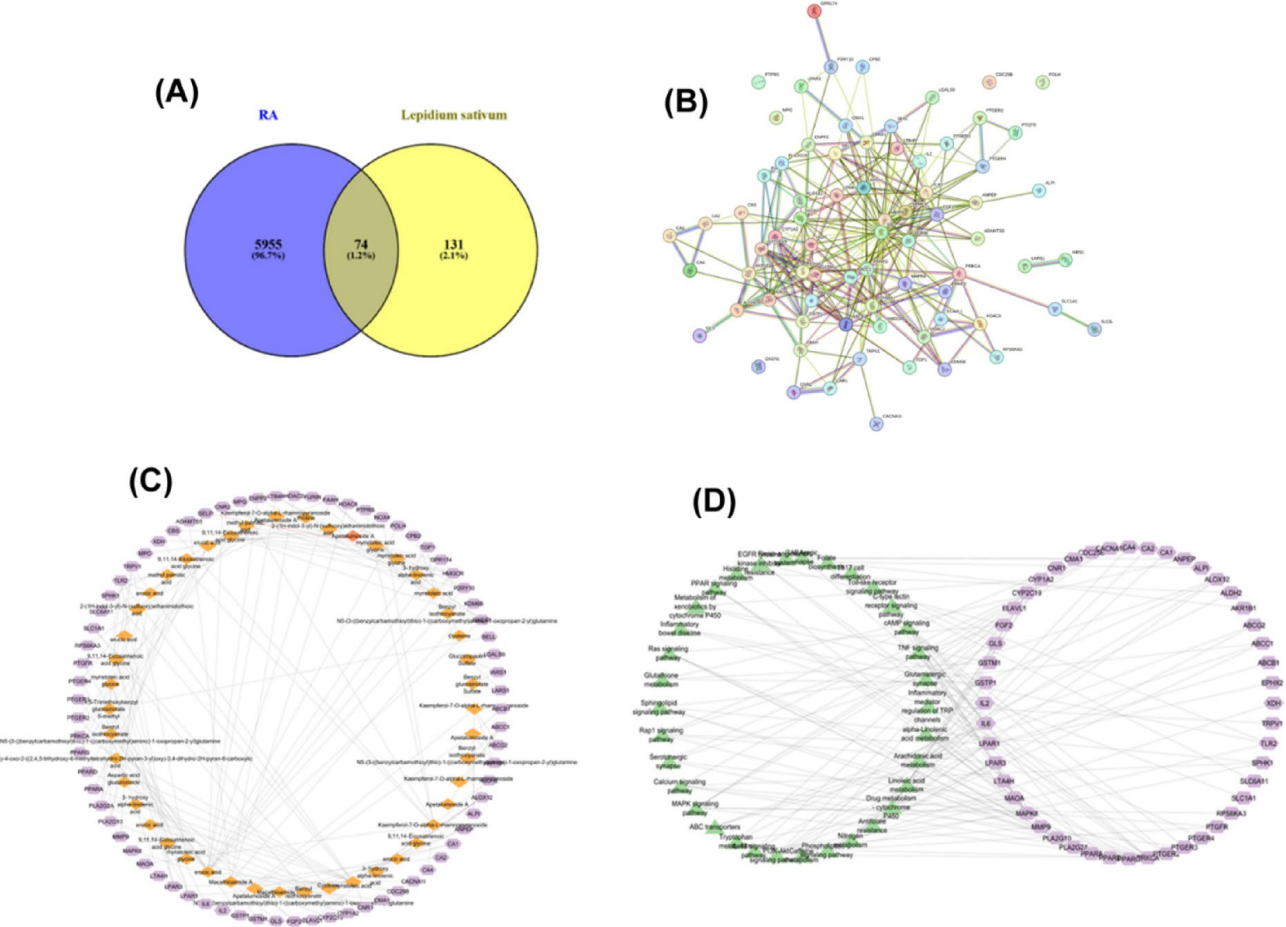
**(A)** Venn diagram showing the common genes between *L. sativum* and RA. **(B)** Protein–protein interactions (PPIs) network of key targets related to RA from STRING database. **(C)** *L. sativum* absorbable compounds-targets network; the orange diamonds reflect the blood migrating components while the violet hexagons represent the target genes related to RA, and the grey lines represent the interactions between the compounds and the genes. **(D)** KEGG pathways—targets network, the violet hexagons represent the target genes related to RA while the green triangles represent the related pathways.

To provide a more comprehensive scientific understanding of *Lepidium sativum*'s mechanism of action against rheumatoid arthritis (RA), a Venn diagram (Fig. 2A) illustrates the overlap between RA-related genes, sourced from GeneCards (http://genecards.org/), and the genes interacting with the 26 metabolites identified in the *Lepidium* extract based on its *in-vivo* metabolic profile. These blood-absorbed metabolites were used as probes to predict related gene targets using databases such as STITCH, SEA, and SwissTargetPrediction. A total of 467 targets were identified from these analyses. The UniProt database was subsequently utilized to filter for human-specific gene names, which are listed in Table S2.

To evaluate the molecular interactions between the absorbed compounds and their targets, a "combined score" was assigned, with higher scores indicating stronger interactions. Genes with a combined score > 0.4 were selected as principal targets. The predicted target genes were analyzed through the STRING database to construct a protein–protein interaction (PPI) network, revealing the functional interactions between the targets. The PPI network (Fig. 2B) consisted of 74 nodes representing biologically significant proteins and 263 edges indicating protein–protein interactions. The average node degree was 7.11, with a PPI enrichment *p*-value < 1.0e-16, highlighting the robustness and biological significance of the interactions.

Additionally, this combined data underscores the potential of *Lepidium sativum* metabolites to modulate complex molecular pathways, offering a promising avenue for future drug discovery in RA management. The resulting integrated dataset was analyzed using Cytoscape 3.10.1, which generated a component-target network (Fig. 2C). This network comprised 119 nodes and 152 edges, including 46 absorbed compounds and 73 identified targets. On average, each compound interacted with approximately 3.434 targets, demonstrating the potential for multiple drug-target interactions, a key attribute for addressing complex multifactorial diseases like RA.

In the degree analysis, the top ten potential targets closely associated with rheumatoid arthritis (RA) pathways and identified in the context of the 26 blood-absorbed metabolites of *Lepidium sativum* were: 3-hydroxy-3-methylglutaryl-coenzyme A reductase (HMGCR), fatty-acid amide hydrolase 1 (FAAH), cannabinoid receptors 1 (CNR1) and 2 (CNR2), transient receptor potential cation channel subfamily V member 1 (TRPV1), toll-like receptor 2 (TLR2), oxoeicosanoid receptor 1 (OXER1), lysophosphatidic acid receptor 1 (LPAR1), arachidonate 12-lipoxygenase, 12S-type (ALOX12), and group 10 secretory phospholipase A2 (PLA2G10). The distribution percentage of top 20 potential targets affected by *L. sativum* absorbed serum metabolites is shown as Fig. S2.

The key bioactive components targeting these pathways synergistically, with significant degree centrality, included myristoleic acid, myristoleic acid glycine, erucic acid (a metabolite of nervonic acid), methyl isoheptadecanoate, 9,11,14-eicosatrienoic acid glycine, 3-hydroxy-alpha-linolenic acid, and benzyl isothiocyanate. Figure S3 shows the distribution percentage of serum metabolites after *L. sativum* ethanolic extract administration. Erucic acid and myristoleic acid with 19% and 14%, respectively.

Previous studies have demonstrated that polyunsaturated fatty acids (PUFAs) such as alpha-linolenic acid and 9,11,14-eicosatrienoic acid effectively suppress proinflammatory cytokines, including TNF-α, interleukin-6 (IL-6), and IL-1β, through direct inhibition of NF-κB and MAPKs pathways^49^. Additionally, a cohort study involving 32,000 women with a mean follow-up of 7.5 years identified that a dietary intake of PUFAs exceeding 0.21 g/day was associated with a 35% reduced risk of developing RA, compared to lower intake levels, during which 205 RA cases were reported^50^.

The identification of PUFA compounds, including alpha-linolenic acid and 9,11,14-eicosatrienoic acid, among the serum-absorbed metabolites of *Lepidium sativum*, is particularly noteworthy. These findings are consistent with prior cohort studies demonstrating an inverse association between dietary PUFA intake and RA risk. For instance, prospective analyses from the Nurses' Health Study indicated that higher intake of omega-3 PUFAs was associated with a reduced incidence of RA, largely attributed to their anti-inflammatory effects via modulation of eicosanoid synthesis and cytokine production^51^.

Moreover, clinical supplementation trials with PUFAs such as eicosapentaenoic acid (EPA) and docosahexaenoic acid (DHA) have demonstrated improvements in disease activity scores (DAS28) and reductions in joint swelling and pain^52^. Although alpha-linolenic acid must be enzymatically converted into EPA/DHA, it still exerts direct anti-inflammatory actions by serving as a substrate for less inflammatory prostaglandins and leukotrienes^53^. The detection of such bioavailable PUFAs in our study supports the hypothesis that *Lepidium sativum* may mediate part of its anti-RA effects through lipid metabolism regulation and attenuation of inflammatory cascades. This aligns our findings with epidemiological and clinical evidence underscoring the therapeutic potential of dietary PUFAs in RA management.

Moreover, recent study has proved that glucosinolates as glucoraphanin and glucobrassicin have an anti-inflammatory effect and improvement of bone health^54^.

FAAH (Fatty-acid amide hydrolase 1), CNR1 (Cannabinoid receptor 1), CNR2 (Cannabinoid receptor 2), HMGCR (3-hydroxy-3-methylglutaryl-coenzyme A reductase), and TRPV1 (Transient receptor potential cation channel subfamily V member 1) have been identified as key targets with critical roles in the pathogenesis of rheumatoid arthritis (RA). FAAH, a membrane-bound enzyme, regulates the biological activity of endocannabinoids such as anandamide and 2-arachidonoylglycerol (2-AG), which are lipid molecules known to modulate inflammation, pain, and neuroprotective effects through interactions with cannabinoid receptors^55^. Depending on the receptor subtype and signaling pathways involved, endocannabinoids can exert either pro-inflammatory or anti-inflammatory effects^56^. FAAH is able to hydrolyze endocannabinoids (EC) as anandamide and 2-AG which are identified and distinguished at ratable levels in the synovial fluid from patients with rheumatoid arthritis (RA) and osteoarthritis (OA) while being absent in healthy persons. FAAH hydrolyzes endocannabinoids, including anandamide and 2-AG, which have been detected at significant levels in the synovial fluid of patients with RA and osteoarthritis (OA) but are absent in healthy individuals^56^. Recent research underscores the central role of FAAH in RA pathology, highlighting that FAAH inhibition increases endocannabinoid levels, thereby enhancing their anti-inflammatory effects. This suggests a promising therapeutic approach for mitigating RA symptoms. However, despite extensive efforts to develop effective FAAH-targeted drugs for clinical use, progress has been hindered by the enzyme's structural complexity and the challenges associated with designing selective inhibitors^55^. These findings emphasize the need for further research into FAAH as a therapeutic target in RA management. A study has confirmed that the CNR1 and CNR2 genes, responsible for the expression of cannabinoid CB1 and CB2 receptors, respectively, along with their associated endocannabinoids, are present in the synovial tissues of late-stage rheumatoid arthritis (RA) patients. Elevated levels of anandamide (AEA) and 2-arachidonoylglycerol (2-AG) in the synovial fluid of patients with RA and osteoarthritis (OA), compared to non-inflamed controls, indicate a significant functional role of the endocannabinoid receptor system in these conditions. This suggests that the cannabinoid receptor system is a critical therapeutic target for managing pain and inflammation associated with RA^57^.

HMGCR (3-hydroxy-3-methylglutaryl-coenzyme A reductase), the enzyme responsible for converting HMG-CoA to mevalonic acid, represents the rate-limiting step in cholesterol biosynthesis (Friesen and Rodwell, 2004). Recent studies have demonstrated that HMGCR inhibitors, such as statins, play a significant role in autoimmune diseases, particularly RA, by exerting anti-inflammatory effects through controlling the pro-inflammatory IL-1β, IL-18, IL-23, and anti-inflammatory IL35^58^ and alleviating RA symptoms^59^ as statins have a proved role in suppression of osteoblast apoptosis and blockage of osteoclastogenesis^60^ .

TRPV1 (Transient receptor potential cation channel subfamily V member 1), a calcium-permeable channel primarily associated with pain perception, has also been implicated in immunological responses in RA. Research has shown that synovial fibroblasts (SF) from RA patients express TRPV1, highlighting its involvement in RA pathogenesis^61^. TRPV1 activation mediates the production of pro-inflammatory cytokines and chemokines, such as IL-6, which contribute to joint pain, inflammation, and destruction. Furthermore, TRPV1 activation enhances IL-8 levels through MAPK and NF-κB pathways, while promoting the overproduction of IL-17, TNF-α, and IFN-γ, which drive inflammatory responses and osteoclastogenesis. These findings underscore the multifaceted role of TRPV1 in the persistence and progression of RA^62^. Collectively, the evidence highlights the pivotal roles of CNR1, CNR2, HMGCR, and TRPV1 in the pathogenesis of RA, making them valuable targets for therapeutic intervention. It is noteworthy that TRPV1 activation plays a pivotal role in mediating the production of pro-inflammatory cytokines and chemokines, such as IL-6, which significantly contribute to joint pain, inflammation, and tissue destruction in rheumatoid arthritis (RA) patients. Elevated levels of TRPV1 activate key signaling pathways, including MAPK and NF-κB, leading to an increase in the chemokine IL-8, which amplifies the inflammatory response. Additionally, the overproduction of cytokines such as IL-17, TNF-α, and IFN-γ triggers a cascade of inflammatory processes that promote osteoclastogenesis, further exacerbating joint damage and bone resorption through TRPV1 involvement^61^.

Among the key targets identified, 3-hydroxy-3-methylglutaryl-CoA reductase (HMGCR) and transient receptor potential vanilloid 1 (TRPV1) play notable roles in RA pathophysiology. HMGCR, the rate-limiting enzyme in cholesterol biosynthesis, has been implicated in promoting systemic inflammation through lipid dysregulation pathways, thereby aggravating autoimmune conditions like RA. Inhibition of HMGCR (e.g., via statins) has shown anti-inflammatory benefits in RA patients by reducing pro-inflammatory cytokine production and joint damage^61^. Activation of TRPV1 contributes to synovial hyperplasia, inflammatory cell infiltration, and pain hypersensitivity, whereas its inhibition has been shown to ameliorate joint inflammation and nociception in experimental RA models.

These findings support the potential therapeutic significance of *Lepidium sativum*-derived metabolites targeting HMGCR, TRPV1, and other pivotal mediators involved in inflammation, cartilage degradation, and immune regulation in rheumatoid arthritis.

#### Prediction of the important signaling pathways of *L. sativum* for treatment of RA

**Fig. 3:**
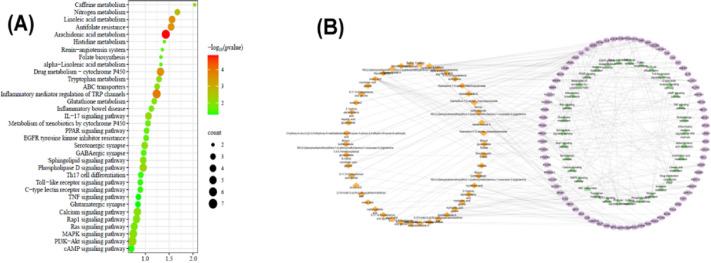
(**A**) The top 20 KEGG signaling pathways based on enrichment score. (**B**) The Component-Target- Pathway network.

To elucidate the mechanisms underlying the anti-arthritic efficacy of *Lepidium sativum* components in plasma, a KEGG pathway enrichment analysis was conducted on the 74 identified targets. This analysis enabled the construction of a network diagram mapping genes to signalling pathways, highlighting significant pathways involved in rheumatoid arthritis (RA) pathogenesis. As depicted in Fig. 2D and detailed in Table S3, a total of 64 KEGG pathways with a false discovery rate (FDR) < 0.05 were identified. The top 20 enriched signalling pathways, shown in Fig. 3A, include pathways such as arachidonic acid metabolism, inflammatory regulator modulation of TRP channels, linoleic acid metabolism, and the antifolate resistance pathway, all of which are closely linked to the pathological processes of RA.

The analysis revealed that *L. sativum* components exert their anti-arthritic effects by synergistically modulating multiple pathways, as illustrated in Fig. 2D. The arachidonic acid metabolism pathway, for instance, plays a pivotal role in regulating inflammatory bone diseases such as RA. This pathway influences a series of reactions in T-cells and associated mediators, leading to heightened synovial inflammation^63^. Furthermore, it is primarily responsible for the production of metabolites such as prostaglandins (PGs), leukotrienes (LTs), thromboxanes (TXs), lipoxins, and hydroxyeicosatetraenoic acids (HETEs). These metabolites drive the destruction of cartilage and bone by activating pro-inflammatory cytokines such as TNF-α, IL-1, and IL-6, as well as chemokines like IL-8. These mediators initiate a cascade of inflammatory actions, culminating in the generation of osteoclasts, joint and cartilage destruction, and eventually leading to joint stiffness and deformity^64^.

Among the most significantly enriched pathways, the inflammatory regulator modulation of TRP channels pathway plays a pivotal role in the pathogenesis of rheumatoid arthritis (RA). Activation of TRPV1 in the synovial fibroblasts (SF) of RA patients has been shown to induce the generation of reactive oxygen species (ROS) and increase the production of inflammatory mediators such as prostaglandins (PGs), IL-6, and IL-8. These mediators stimulate hyperplasia of synoviocytes and contribute to tissue degradation^65^.

Furthermore, recent studies have demonstrated that the linoleic acid metabolism pathway exacerbates the pro-inflammatory state of SF in RA. Free saturated and unsaturated fatty acids have been found to elevate the secretion of pro-inflammatory mediators, including IL-6, IL-8, MCP-1, pro-MMP1, and MMP3, which are implicated in joint inflammation and degradation.

Additionally, folic acid (FA) oxidation has been shown to provoke osteoclast precursor (OCP) adhesion and stimulate joint destruction in RA^66^. The antifolate resistance pathway also plays a critical role in RA, particularly concerning methotrexate resistance (MTXR). Methotrexate, an antifolate drug targeting enzymes involved in DNA biosynthesis, is widely used in RA management. However, recent studies have revealed that inflammatory cytokines, ABC transporters, T-cells, interleukins, TNF-α, and other mediators contribute to the development of MTXR. Molecular signaling pathways, including the NF-κB pathway, GPCR pathway, and folate-dependent metabolic pathways, further influence MTXR through their regulatory roles in RA^67^. Figure 3B illustrates the network connecting *Lepidium sativum* metabolites, their molecular targets, and the associated pathways, providing a comprehensive view of the mechanisms through which these metabolites mitigate RA pathogenesis. This integrative network highlights the complex interplay between metabolic pathways and inflammatory processes in RA, emphasizing the multi-target therapeutic potential of *L. sativum*.

#### Effects of *L. sativum* administration on ankle diameter, arthritic score and body weight

**Fig. 4:**
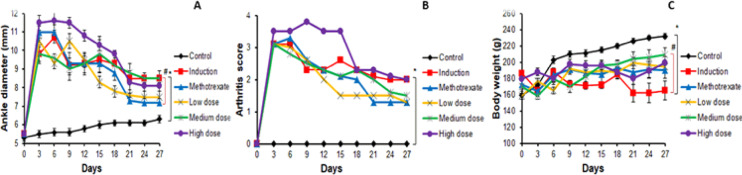
Effects of *L. sativum* administration on ankle diameter (**A**), arthritis score (**B**) and body weight (**C**) of RA rats from day 0 to day 27. *P < 0.05 induction group *versus* control group, #*P* < 0.05 treatment groups *versus* induction group.

The therapeutic effects of *Lepidium sativum* administration were evaluated by monitoring ankle diameters, arthritis scores, and body weights of rats throughout the experimental period, from day 0 to day 27. As illustrated in Fig. 4A,B, the ankle diameters and arthritis scores in all RA groups showed a significant increase from day 3 onwards compared to the control group. By day 27, methotrexate treatment significantly reduced ankle diameters compared to the induction group. Additionally, treatment with either methotrexate or *L. sativum* (low and medium doses) reduced arthritis scores to levels that were not statistically different from the control group.

Body weight changes were also tracked to assess systemic effects. By the end of the study, rats in the induction group exhibited significant body weight loss compared to the control group, as shown in Fig. 4C. Notably, only treatment with *L. sativum* at a medium dose significantly improved body weights compared to the induction group.

Overall, methotrexate effectively alleviated arthritis symptoms, as evidenced by reductions in ankle diameter and arthritis scores. However, these benefits were not accompanied by restoration of body weight, highlighting potential systemic toxicity. Conversely, *L. sativum* at a medium dose improved arthritis scores without significantly reducing paw diameter but demonstrated a significant positive effect on body weight. This suggests that *L. sativum* may offer a more holistic therapeutic benefit by mitigating arthritis symptoms while simultaneously addressing systemic health indicators such as body weight.

#### Effects of *L. sativum* administration on RA progression using morphological analysis

**Fig. 5:**
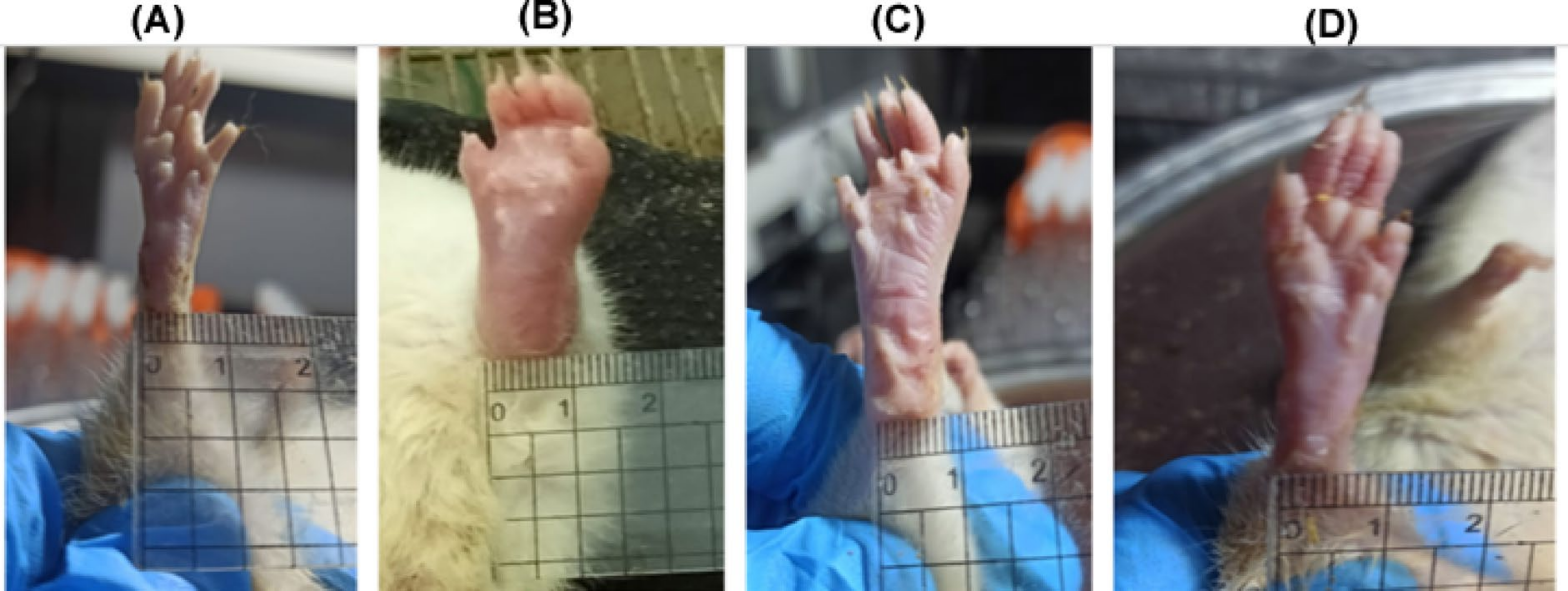
Effects of *L. sativum* administration on the macroscopic features of the left hind paw of RA rats. **(A)** Control group, **(B)** Induction group, **(C)** *L. sativum* treatment group and **(D)** Methotrexate group.

By the end of the experiment, distinct morphological differences in the left hind paw were observed among the groups. The pathological signs were entirely absent in the control group (Fig. 5A). In the induction group (Fig. 5B), the paws exhibited pronounced swelling, redness, and stiffness, indicative of severe rheumatoid arthritis (RA). Treatment with either *Lepidium sativum* or methotrexate alleviated the visible symptoms of RA, with methotrexate demonstrating superior efficacy (Fig. 5C,D, respectively). These findings further support the therapeutic potential of *L. sativum* in mitigating RA symptoms, albeit to a lesser extent compared to methotrexate.

#### Effects of *L. sativum* administration on organ index and liver enzymes

**Fig. 6:**
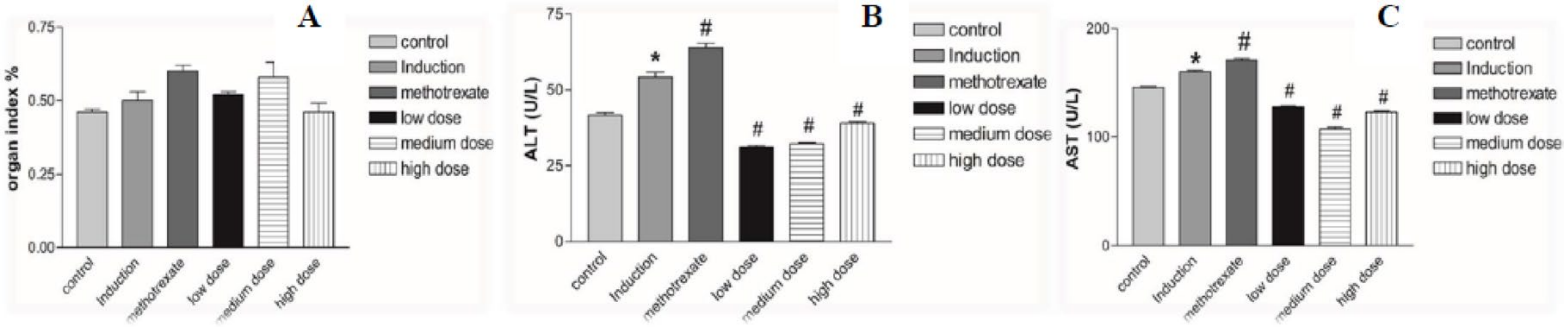
Effects of *L. sativum* administration on **(A)** organ index, **(B)** serum ALT level, and **(C)** serum AST level in RA rats. *P < 0.05 induction group versus control group, #*P* < 0.05 treatment groups versus induction group.

The organ indices for the spleen and thymus, as well as liver enzyme levels, were assessed to evaluate potential toxic effects of the treatments. As shown in Fig. 6A, treatment with either methotrexate or *Lepidium sativum* did not result in significant changes in organ indices compared to the control or induction groups.

However, liver enzyme analysis revealed contrasting effects of the treatments on hepatic health. As depicted in Fig. 6B,C, the levels of ALT and AST were significantly elevated in the induction group compared to the control group, indicating liver damage associated with rheumatoid arthritis (RA). These enzyme levels were further exacerbated by methotrexate treatment, suggesting potential hepatotoxicity. In contrast, treatment with *L. sativum* significantly reduced ALT and AST levels compared to both the induction group and the control group, demonstrating its hepatoprotective properties.

These findings highlight the biosafety of *L. sativum* relative to conventional RA treatments like methotrexate. While methotrexate effectively alleviates RA symptoms, its systemic toxicity, particularly hepatotoxicity, underscores the importance of exploring alternative treatments such as *L. sativum*, which offers both therapeutic efficacy and a favourable safety profile.

#### Effects of *L. sativum* on serum biochemical indices

**Fig. 7:**
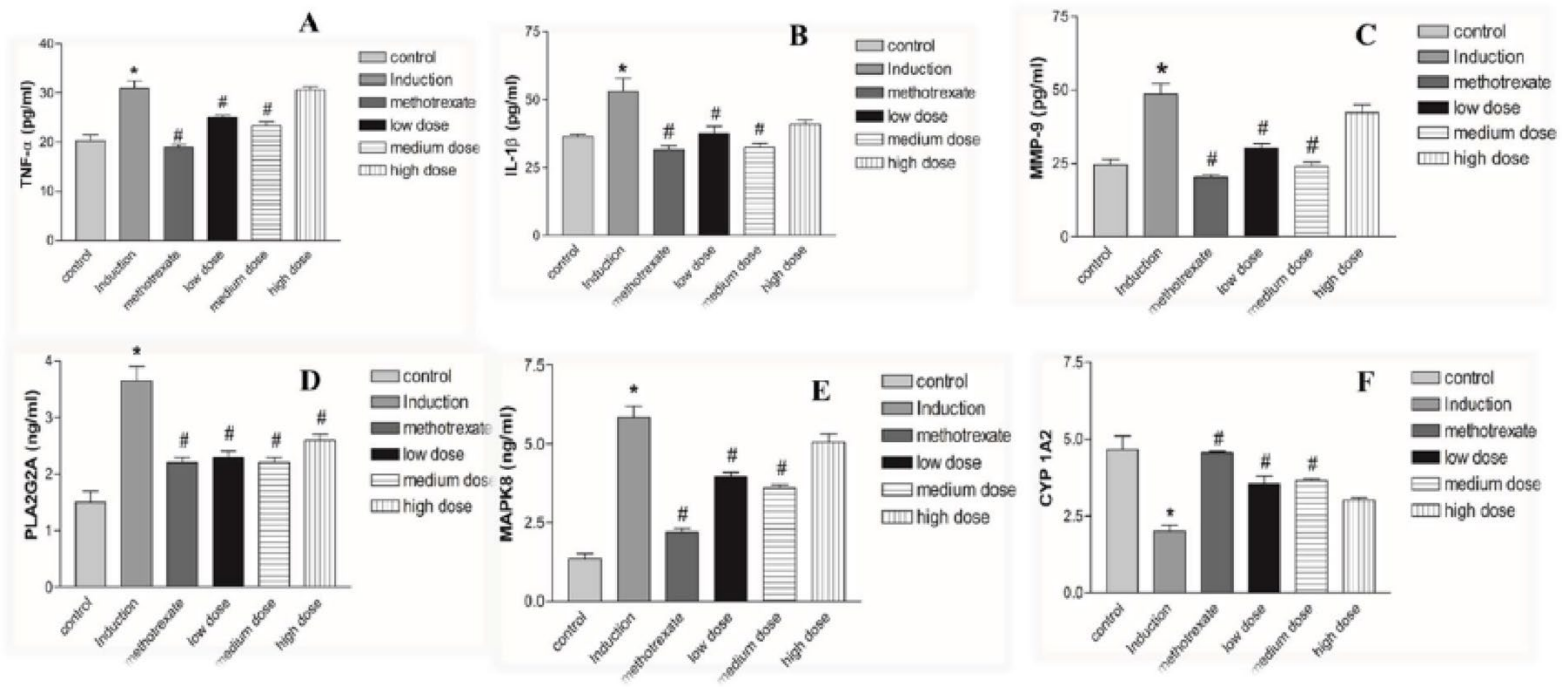
Effects of *L. sativum* administration on **(A)** serum Levels of TNF-α, **(B)** IL-1β, **(C)** MMP-9, **(D)** PLA2G2A, **(E)** MAPK8 and **(F)** CYP 1A2 in RA rats. *P < 0.05 induction group versus control group, #*P* < 0.05 treatment groups versus induction group.

Serum levels of TNF-α, IL-1β, and MMP-9 were measured using commercially available ELISA kits. The induction group showed significantly elevated levels of inflammatory cytokines TNF-α, IL-1β, and MMP-9 compared to the control group, as depicted in Fig. 7A–C, respectively. Treatment with methotrexate (MTX) or *Lepidium sativum* (low and medium doses) significantly reduced TNF-α and IL-1β levels compared to the induction group. Similarly, MMP-9 levels, which were markedly elevated in the induction group, were significantly decreased following treatment with either MTX or *L. sativum* at both doses.

These findings collectively suggest that *L. sativum* administration effectively suppresses inflammation by reducing pro-inflammatory cytokines and MMP-9, thereby mitigating RA progression. The results highlight *L. sativum* as a promising therapeutic candidate with anti-inflammatory properties, comparable to MTX, but with a potentially safer profile.

#### Validation of anti-RA mechanism of *L. sativum*

At a detailed level of investigation, KEGG pathway analysis identified **CYP1A2**, **PLA2G2A**, and **MAPK8** as the top potential targets, primarily clustered within key inflammatory pathways, including the **arachidonic acid metabolism pathway**, **inflammatory regulator modulation of TRP channels pathway**, **linoleic acid metabolism pathway**, and **antifolate resistance pathway**. These findings provided a rationale for measuring these biochemical markers using the ELISA technique.

**Cytochrome P450 enzymes (CYP1A2)** play a critical role in drug metabolism and have been implicated in the metabolism of polyunsaturated fatty acids (PUFAs) such as linoleic acid. However, CYP1A2 activity is known to be suppressed by inflammatory cytokines and interferons, which are prevalent in inflammatory conditions such as rheumatoid arthritis (RA). Furthermore, elevated cellular levels of PUFAs can regulate CYP enzyme activity, suggesting a feedback mechanism that influences inflammatory and metabolic processes. These insights emphasize the interconnected roles of CYP1A2, PLA2G2A, and MAPK8 in the inflammatory pathways associated with RA progression and highlight their potential as therapeutic targets^68^. Furthermore, linoleic acid is exposed to series of reactions inside the body transforming to other forms as arachidonic acid and deposited in cell membrane as phospholipids. As inflammation takes place, the PUFAs fused into cell membrane can be set free by phospholipase A2 and being as substrates for oxidation by cyclo-oxygenase, lipo-oxygenase, and cytochrome P450 enzymes (CYPs) to form prostaglandins, thromboxanes and leukotrienes^69^. Further, Phospholipase A2GIIA (PLA2G2A) is involved in arachidonic acid metabolism and linoleic acid metabolism pathways as studies proved that it was found with significant increase in plasma in inflammatory diseases such as rheumatoid arthritis. Also, disarrangement of cell membranes by phospholipid scramblase elevates PLA2G2A activity and arachidonic acid release which can be handled by cyclo-oxygenase (COX) and lipo-oxygenase (LOX) to form eicosanoids with prostaglandins and leukotrienes(LT)^70^. As well, Mitogen activated protein kinase (MAPK) has a main role in inflammatory regulator modulation of TRP channels pathway as it was illustrated that elevated levels of TRPV1 in the hyperosmotic condition and response to intense temperature changes stimulate MAPK and NF-kB activation and facilitate an increase in the chemokine IL-8 which in turn leads to inflammation and joint destruction^61^.

Experimentally speaking, Phospholipase A2, Group IIA (PLA2G2A) and Mitogen-activated protein kinase 8 (MAPK8) protein levels were significantly increased in the induction group compared to control group. PLA2G2A levels were significantly reduced in all treatment groups (MTX or *L. sativum*) compared to induction while MAPK8 levels were significantly reduced by all treatments except *L. sativum* (high dose). On the contrary, Cytochrome P450 1A2 (CYP 1A2) levels were significantly reduced in the induction group compared to control group and its levels were significantly restored by treatment with either MTX or *L. sativum* (low dose and medium dose) (Fig. 7D–F). These results indicated that *L. sativum* blocked the main pathways involved in the inflammatory process of RA.

#### Histopathological examination

**Fig. 8:**
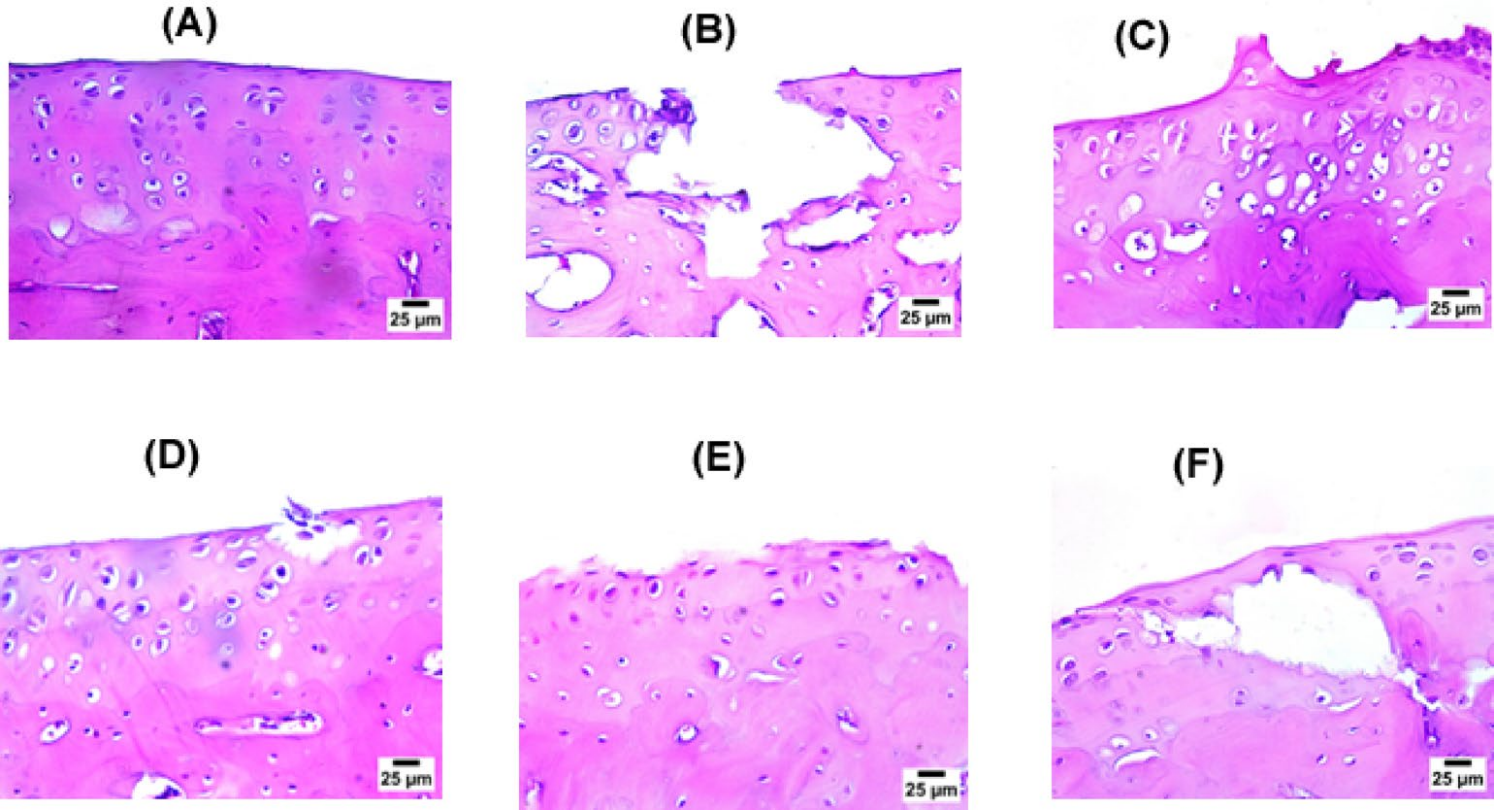
Photomicrographs of hematoxylin & eosin (H&E) stained sections of ankle joints of rats in all treatment groups. Control group **(A)** showing normal histological structure of articular surface, induction group **(B)** showing complete destruction and roughness in articular surface, methotrexate group **(C)** showing roughness of articular surface, *L. sativum* (low dose) **(D)** showing mild destruction and roughness in articular surface, *L. sativum* (medium dose) **(E)** showing roughness of articular surface, and *L. sativum* (high dose) **(F)** showing partial destruction in articular surface.

**Fig. 9:**
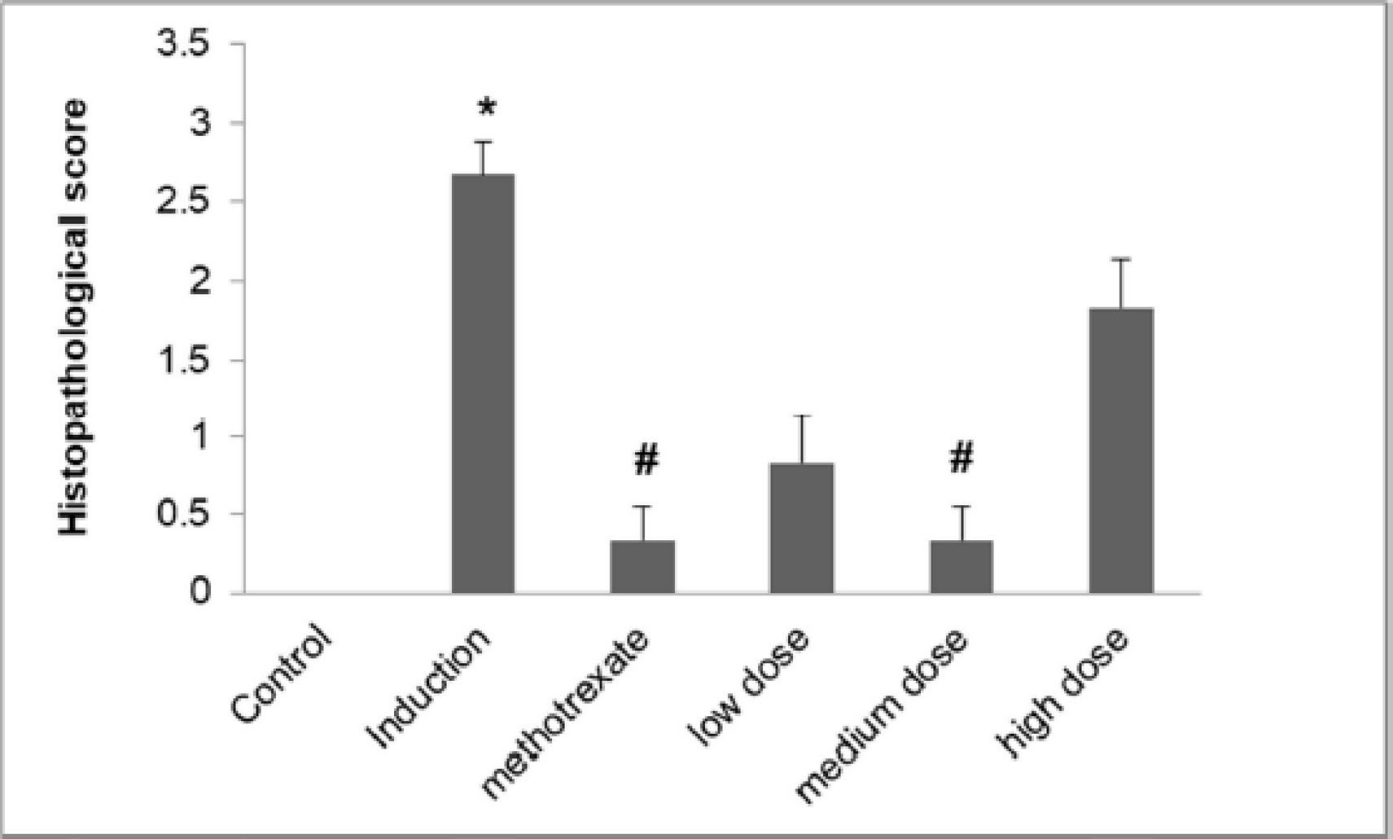
Effects of *L. sativum* administration on histopathological score. *P *<* 0.05 induction group *versus* control group, #P *<* 0.05 treatment groups *versus* induction group.

Examination of hematoxylin & eosin (H&E) stained sections of ankle joints emphasized the protective effect of *L. sativum* against adjuvant-induced arthritis (Fig. 8). Sections of control group showed normal histological structure of articular surface with mean score 0 (Fig. 8A) while there was a complete destruction and roughness in articular surface in the induction group with mean score 2.7 ± 0.2 (Fig. 8B). Treatment with either MTX or *L. sativum* reduced joint damage. In MTX group, only roughness of articular surface was detected with mean score 0.3 ± 0.2 (Fig. 8C). For *L. sativum*, medium dose caused the highest recovery where no destruction was noted in the articular surface with mean score 0.3 ± 0.2 (Fig. 8E). Figure 9 shows the effect of *L. sativum* on histopathological score. All these results indicating that *L. sativum* could be used as a co-therapy to alleviate the symptoms of RA and protect the patients from further complications.

## Conclusion

In conclusion, the present study represents a significant effort to explore the efficacy of *Lepidium sativum* in managing rheumatoid arthritis (RA) by integrating serum pharmacochemistry and network pharmacology. Twenty-six serum-circulating compounds, including eleven prototypes and fifteen metabolites, were identified and characterized as potential bioactive constituents. These compounds were effectively absorbed and detected at significant concentrations in *L. sativum*-treated sera, demonstrating their potential to alleviate the inflammatory processes associated with RA.

The network pharmacology analysis revealed that the primary mechanisms underlying the anti-arthritic effects of *L. sativum* involve targeting key molecules such as FAAH, CNR1, CNR2, HMGCR, and TRPV1, alongside the regulation of critical pathways including the arachidonic acid metabolism pathway, inflammatory regulator modulation of TRP channels pathway, linoleic acid metabolism pathway, and antifolate resistance pathway. These pathways are intricately linked to synovial inflammation and bone damage in RA pathophysiology.

Furthermore, experimental findings validated the predictions of network pharmacology, highlighting the significant anti-inflammatory effects of *L. sativum* through the down-regulation of inflammatory mediators such as **TNF-α**, **IL-1β**, **MMP-9**, **CYP1A2**, **PLA2G2A**, and **MAPK8**. These results underscore the potential of *L. sativum* as a therapeutic agent with a multifaceted mechanism of action for mitigating RA.

This study provides a novel perspective for systematically investigating the efficacy and mechanisms of *L. sativum*, offering a foundation for its application in addressing the complex pathophysiology of RA. Future research should focus on further validating these findings with robust clinical evidence to expand therapeutic strategies in the era of personalized medicine. Achieving a comprehensive “treat-to-target” approach could hold significant implications for improving clinical outcomes in RA management.

## Supplementary Information

Below is the link to the electronic supplementary material.

Supplementary Material 1

## Data Availability

Data is provided within the manuscript or supplementary information files.

## Declarations

### KEGG permission

If you do not use KEGG database IMAGE(S) in your paper, there is no need to obtain copyright permission from us. However, since you are utilizing KEGG in your research, we kindly ask you to cite KEGG paper(s) (www.kegg.jp/kegg/kegg1.html).
