# Supplementary material for: Serum metabolomics-driven network pharmacology elucidate the anti-rheumatoid arthritis potential of garden cress
Source: Sci Rep. 2025 Sep 1;15:32091. doi: 10.1038/s41598-025-13412-6 (PMC12402230; doi:10.1038/s41598-025-13412-6)
Supplement: Supplementary file 1 — Supplementary Material 1 [file 41598_2025_13412_MOESM1_ESM.docx]

# **UPLC- MS/MS parameters and conditions**

The UPLC-MS/MS was performed using Waters Corporation, Milford, MA01757, U.S.A. The chromatographic separation was performed using the Waters ACQUITY UPLC® BEH C18 column, with dimensions of 50 mm × 2.1 mm ID × 1.7 μm particle size. The column was operated at a flow rate of 0.2 ml.min^-1^ and a temperature of 30°C. The analysis was conducted using a gradient elution method, where the concentration of phase B was increased. A binary mobile phase consisting of 0.1% formic acid in ultrapure water (Phase A) and 0.1% formic acid in acetonitrile (Phase B) was utilized. As the sets for the elution gradient were automated as follows: 99% (phase A), 1 % (phase B) at retention time of 0.0–04.0 min, 90% (phase A), 10% (phase B) at 04.0–07.0 min, 80% (phase A), 20% (phase B) at 07.0–09.0 min, 70% (phase A), 30% (phase B) at 09.0–15.0 min, 30% (phase A), 70% (phase B) at 15.0–26.0 min, 100 % (phase B) at 26.0-32.0 min and washing up the step was accomplished at 32.0 min. The sample solution was prepared using UPLC analytical grade methanol at a concentration of 1 mg/ml. The filtration process involved the use of a membrane disc filter with a pore size of 0.2 μm. Prior to injection, the sample was subjected to sonication in order to remove any trapped gases. Subsequently, a volume of 2 μl was injected into the UPLC apparatus. The working conditions of ESI were optimized by adjusting several parameters included the capillary voltage set at 3 kV, the temperature of the ion source maintained at 150 ^◦^C, the cone voltage set to 30 V, the pressure of the nitrogen gas nebulizer set at 35 psi, and the temperature of the drying and sheath gas set at 400 ^◦^C. The optimal flow rates for the sheath and drying gas were determined to be 50 L/h and 600 L/h, respectively. The duration of the analytical run was increased to 30 minutes.

**Table S1: Metabolites identiﬁed in *L. sativum* extract using UPLC-ESI-MS/MS in both negative and positive ionization modes**

| **No.** | **Rt (min.)** | **Identiﬁed compounds** | **Precursor ions** | **Molecular Formula** | **MS/MS Product Ions** | **Chemical class** | Related reference |
| --- | --- | --- | --- | --- | --- | --- | --- |
| 1 | 0.09 | Valine | [M+H] ^+^  118.15 | C_5_H_11_NO_2_ | 74 | Amino acid | (1) |
| 2 | 0.75 | Cysteine | [M+H] ^+^  122.16 | C_3_H_7_NO_2_S | 78 | Amino acid | (1) |
| 3 | 0.78 | Hexose | [M+H] ^+^  181.16 | C_6_H_12_O_6_ | 131, 120, 103 | Monosaccharide | (2) |
| 4 | 0.80 | Glycine | [M+H] ^+^  76.07 | C_2_H_5_NO_2_ | 32 | Amino acid | (1) |
| 5 | 0.90 | Aspartic acid | [M-H] ^_^  132.09 | C_4_H_7_NO_4_ | 88 | Amino acid | (1) |
| 6 | 0.92 | Niacin | [M+H] ^+^  124.11 | C_6_H_5_O_2_N | 80 | Amino acid | (3) |
| 7 | 1.23 | Serine | [M+H] ^+^  106.09 | C_3_H_7_NO_3_ | 62 | Amino acid | (1) |
| 8 | 1.49 | Proline | [M+H] ^+^  116.13 | C_5_H_9_NO_2_ | 72 | Amino acid | (1) |
| 9 | 2.02 | Semilepidinoside A | [M+H] ^+^  337.35 | C_16_H_20_N_2_O_6_ | 175 | Glycoalkaloid | (4) |
| 10 | 2.02 | Rosmarinic acid | [M-H]^-^  359.29 | C_18_H_16_O_8_ | 197, 179 | Phenolic acid | (5) |
| 11 | 5.40 | Glucotropeolin | [M+H] ^+^  410.40 | C_14_H_19_NO_9_S_2_ | 327, 309, 224, 165 | Glucosinolate | (6) |
| 12 | 5.82 | Benzyl glucosinolate | [M-H]^-^  408.42 | C_14_H_18_NO_9_S_2_ | 276, 242 | Glucosinolate | (7) |
| 13 | 6.50 | Apetalumoside A | [M-H]^-^  801.67 | C_34_H_42_O_22_ | 639, 477, 315 | Flavonoid glycoside | (8) |
| 14 | 6.94 | Macaridine | [M+H] ^+^  216.25 | C_13_H_13_NO_2_ | 198, 169, 152 | Alkaloid | (2) |
| 15 | 7.18 | Lepidine B/E/F | [M+H] ^+^  347.39 | C_20_H_18_N_4_O_2_ | 174 | Alkaloid | (4) |
| 16 | 7.24 | Benzyl isothiocyanate | [M+H] ^+^  150.22 | C_8_H_7_NS | 92 | Isothiocyanate | (9) |
| 17 | 7.29 | Acetylbenzylamide | [M+H] ^+^  164.17 | C_9_H_9_NO_2_ | 105, 77 | Amide | (10) |
| 18 | 7.75 | Dithiohexoside | [M-H]^-^  389.42 | C_12_H_22_O_10_S_2_ | 227, 195, 91 | Thioglycoside | (11) |
| 19 | 8.24 | Lepidine AK | [M+H] ^+^  361.42 | C_21_H_20_N_4_O_2_ | 188 | Alkaloid | (4) |
| 20 | 8.25 | Lepidimoic acid | [M-H]^-^  321.26 | C_12_H_18_O_10_ | 276 | Disaccharide | (11) |
| 21 | 9.27 | Macathioamide A | [M-H]^-^  283.37 | C_16_H_16_N_2_OS | 240, 212, 168 | Thioamide | (12) |
| 22 | 9.30 | Kaempferol-7-hexoside | [M+H] ^+^  433.38 | C_21_H_20_O_10_ | 271, 151, 133 | Flavonoid | (5) |
| 23 | 9.56 | Meyeniin C | [M+H] ^+^  309.42 | C_14_H_16_N_2_O_2_S_2_ | 265, 235, 185 | Thiohydantoin | (2) |
| 24 | 9.66 | Apigenin-7-hexoside | [M+H] ^+^  433.40 | C_21_H_20_O_10_ | 271, 227 | Flavonoid glycoside | (5) |
| 25 | 10.12 | Glucobrassicanapin | [M-H]^-^  385.39 | C_12_H_20_NO_9_S_2_- | 97 | Glucosinolate | (13) |
| 26 | 10.34 | Gallocatechin | [M+H] ^+^  307.27 | C_15_H_14_O_7_ | 179, 125 | Flavonoid | (14) |
| 27 | 10.36 | Cyclomethyltryptophan | [M+H] ^+^  217.24 | C_12_H_12_N_2_O_2_ | 144, 113 | Amino acid | (2) |
| 28 | 10.51 | Sinapoyl malate | [M-H]^-^  339.27 | C_15_H_16_O_9_ | 223 | Cinnamic acid derivative | (15) |
| 29 | 10.52 | Glucobrassicin | [M-H]-447.46 | C_16_H_20_N_2_O_9_S_2_ | 367, 205 | Glucosinolate | (16) |
| 30 | 10.84 | Macahydantoin A | [M+H] ^+^  261.35 | C_14_H_16_N_2_OS | 184, 105 | Thiohydantoin | (2) |
| 31 | 11.31 | Macahydantoin C | [M+H] ^+^  247.26 | C_13_H_14_N_2_O_3_ | 170, 107 | Hydantoin derivatives | (2) |
| 32 | 13.29 | Macahydantoin D | [M+H] ^+^  231.26 | C_13_H_14_N_2_O_2_ | 154, 91 | Hydantoin derivatives | (2) |
| 33 | 16.78 | Meyeniihydantoin A | [M+H] ^+^  261.29 | C_14_H_16_N_2_O_3_ | 230, 184, 121 | Hydantoin | (2) |
| 34 | 17.37 | 4-Methoxyglucobrassicin | [M+H] ^+^  479.50 | C_17_H_22_N_2_O_10_S_2_ | 399, 237 | Glucosinolate | (16) |
| 35 | 17.43 | 3,4,5-Trimethoxybenzyl glucosinolate | [M+H] ^+^  500.52 | C_17_H_25_NO_12_S_2_ | 482, 420, 258 | Glucosinolate | (16) |
| 36 | 21.16 | Myristoleic acid | [M-H] ^-^  225.34 | C_14_H_26_O_2_ | 181 | Fatty acid | (17) |
| 37 | 22.87 | Alpha-linolenic acid | [M-H] ^-^  277.40 | C_18_H_30_O_2_ | 233 | Fatty acid | (17) |
| 38 | 31.28 | 9,11,14‑Eicosatrienoic acid | [M-H] ^-^  301.25 | C_20_H_34_O_2_ | 257 | Fatty acid | (17) |
| 39 | 31.45 | Stearic acid | [M-H]-  283.50 | C_18_H_36_O_2_ | 239 | Fatty acid | (17) |
| 40 | 31.45 | Methyl isoheptadecanoate | [M+H]^+^  285.50 | C_18_H_36_O_2_ | 254, 252, 74 | Fatty acid ester | (17) |
| 41 | 31.66 | Nervonic acid | [M-H] ^-^  365.60 | C_24_H_46_O_2_ | 321 | Fatty acid | (17) |

**Table S2: Potential protein targets associated with blood-dissolved *L. sativum* components**

| **Uniport ID** | **Short name of protein** | **Full name of protein** | **Interacting compound (s) (combined interaction score)** |
| --- | --- | --- | --- |
| P05231 | IL6 | Interleukin-6 | 3,4,5-Trimethoxybenzyl glucosinolate S-methyl (0.32) |
| P60568 | IL2 | Interleukin-2 | Apetalumoside A (0.43) |
| P14780 | MMP9 | Matrix metalloproteinase-9 | 2-(1H-indol-3-yl)-N-(sulfooxy) ethanimidothioic acid (0.4) |
| O60603 | TLR2 | Toll-like receptor 2 | myristoleic acid glycine (0.45),9,11,14‑Eicosatrienoic acid glycine (0.41), erucic acid (0.74), myristoleic acid (0.67), methyl isoheptadecanoate (0.54) |
| P05164 | MPO | Myeloperoxidase | 2-(1H-indol-3-yl)-N-(sulfooxy) ethanimidothioic acid (0.41) |
| P45983 | MAPK8 | Mitogen-activated protein kinase 8 | Macathioamide A (0.46) |
| P14555 | PLA2G2A | Phospholipase A2, membrane associated | myristoleic acid glycine (0.54),9,11,14‑Eicosatrienoic acid glycine (0.49), erucic acid (0.48), myristoleic acid (0.44) |
| P14151 | SELL | L-selectin | 3,4,5-Trimethoxybenzyl glucosinolate S-methyl (0.32) |
| P08183 | ABCB1 | ATP-dependent translocase ABCB1 | Kaempferol-7-O-alpha-L-rhamnopyranoside (0.47), lepidine/ AK (0.41) |
| O15496 | PLA2G10 | Group 10 secretory phospholipase A2 | myristoleic acid glycine (0.44),9,11,14‑Eicosatrienoic acid glycine (0.44), erucic acid (0.46), methyl isoheptadecanoate (0.43), myristoleic acid (0.43) |
| P09488 | GSTM1 | Glutathione S-transferase Mu 1 | Benzyl isothiocyanate N5-(3-((benzylcarbamothioyl)thio)-1-((carboxymethyl)amino)-1-oxopropan-2-yl) glutamine (0.52) |
| P09211 | GSTP1 | Glutathione S-transferase P | Benzyl isothiocyanate N5-(3-((benzylcarbamothioyl)thio)-1-((carboxymethyl)amino)-1-oxopropan-2-yl) glutamine (0.63) |
| P37231 | PPARG | Peroxisome proliferator-activated receptor gamma | 9,11,14‑Eicosatrienoic acid glycine (0.65), erucic acid (1), myristoleic acid (0.92) |
| P09038 | FGF2 | Fibroblast growth factor 2 | Aspartic acid glucuronoide (0.4),3-hydroxy-4-oxo-2-((2,4,5-trihydroxy-6-methyltetrahydro-2H-pyran-3-yl) oxy)-3,4-dihydro-2H-pyran-6-carboxylic acid (0.11150186548) |
| P47989 | XDH | Xanthine dehydrogenase/oxidase | Apetalumoside A (0.51), Kaempferol-7-O-alpha-L-rhamnopyranoside (0.69) |
| P33261 | CYP2C19 | Cytochrome P450 2C19 | Macathioamide A (0.5) |
| P35520 | CBS | Cystathionine beta-synthase | Apetalumoside A (0.42), Kaempferol-7-O-alpha-L-rhamnopyranoside (0.47) |
| P05177 | CYP1A2 | Cytochrome P450 1A2 | Macathioamide A (0.41) |
| Q9UNA0 | ADAMTS5 | A disintegrin and metalloproteinase with thrombospondin motifs 5 | Macathioamide A (0.44) |
| P16109 | SELP | P-selectin | myristoleic acid glycine (0.4) |
| Q8NER1 | TRPV1 | Transient receptor potential cation channel subfamily V member 1 | myristoleic acid glycine (0.52),9,11,14‑Eicosatrienoic acid glycine (0.63) (0.57), erucic acid (0.57), myristoleic acid (0.57) |
| O00182 | LGALS9 | Galectin-9 | Glucotropaolin Sulfate (0.3) |
| P35408 | PTGER4 | Prostaglandin E2 receptor EP4 subtype | erucic acid (0.41), methyl isoheptadecanoate (0.41) |
| Q9UNQ0 | ABCG2 | Broad substrate specificity ATP-binding cassette transporter ABCG2 | Kaempferol-7-O-alpha-L-rhamnopyranoside (0.47), Apetalumoside A (0.42) |
| P15144 | ANPEP | Aminopeptidase N | Cysteine (0.44), Benzyl isothiocyanate N5-(3-((benzylcarbamothioyl)thio)-1-((carboxymethyl)amino)-1-oxopropan-2-yl) glutamine (0.42) |
| Q07869 | PPARA | Peroxisome proliferator-activated receptor alpha | erucic acid (1), myristoleic acid (0.92) |
| P33527 | ABCC1 | Multidrug resistance-associated protein 1 | Benzyl isothiocyanate N5-(3-((benzylcarbamothioyl)thio)-1-((carboxymethyl)amino)-1-oxopropan-2-yl) glutamine (0.52), Kaempferol-7-O-alpha-L-rhamnopyranoside (0.47), Apetalumoside A (0.47), |
| P00918 | CA2 | Carbonic anhydrase 2 | Macathioamide A (0.44) |
| Q15717 | ELAVL1 | ELAV-like protein 1 | Kaempferol-7-O-alpha-L-rhamnopyranoside (0.47) |
| P43116 | PTGER2 | Prostaglandin E2 receptor EP2 subtype | erucic acid (0.41), methyl isoheptadecanoate (0.45) |
| P34972 | CNR2 | Cannabinoid receptor 2 | myristoleic acid glycine (0.55),9,11,14‑Eicosatrienoic acid glycine (0.6), erucic acid (0.57), myristoleic acid (0.57) |
| P29372 | MPG | DNA-3-methyladenine glycosylase | Kaempferol-7-O-alpha-L-rhamnopyranoside (0.44) |
| Q9NYA1 | SPHK1 | Sphingosine kinase 1 | erucic acid (0.45), myristoleic acid (0.42) |
| Q13822 | ENPP2 | Ectonucleotide pyrophosphatase/phosphodiesterase family member 2 | erucic acid (0.45), myristoleic acid (0.41) |
| Q92633 | LPAR1 | Lysophosphatidic acid receptor 1 | myristoleic acid glycine (0.55),9,11,14‑Eicosatrienoic acid glycine (0.5), erucic acid (0.5), myristoleic acid (0.47) |
| P05091 | ALDH2 | Aldehyde dehydrogenase, mitochondrial | Kaempferol-7-O-alpha-L-rhamnopyranoside (0.43) |
| Q15722 | LTB4R | Leukotriene B4 receptor 1 | erucic acid (0.55), myristoleic acid (0.51),3- hydroxy alpha-linolenic acid (0.52) |
| P21554 | CNR1 | Cannabinoid receptor 1 | myristoleic acid glycine (0.58),9,11,14‑Eicosatrienoic acid glycine (0.41), erucic acid (0.7),3- hydroxy alpha-linolenic acid (0.49), myristoleic acid (0.58) |
| Q92769 | HDAC2 | Histone deacetylase 2 | Macathioamide A (0.41) |
| P51812 | RPS6KA3 | Ribosomal protein S6 kinase alpha-3 | Kaempferol-7-O-alpha-L-rhamnopyranoside (0.54), Apetalumoside A (0.56) |
| P09958 | FURIN | Furin | Benzyl isothiocyanate N5-(3-((benzylcarbamothioyl)thio)-1-((carboxymethyl)amino)-1-oxopropan-2-yl) glutamine (0.4) |
| P43115 | PTGER3 | Prostaglandin E2 receptor EP3 subtype | erucic acid (0.41) |
| O00519 | FAAH | Fatty-acid amide hydrolase 1 | myristoleic acid glycine (0.76),9,11,14‑Eicosatrienoic acid glycine (0.81), erucic acid (0.63), myristoleic acid (0.58),3- hydroxy alpha-linolenic acid (0.49) |
| P09960 | LTA4H | Leukotriene A-4 hydrolase | Benzyl isothiocyanate N5-(3-((benzylcarbamothioyl)thio)-1-((carboxymethyl)amino)-1-oxopropan-2-yl) glutamine (0.41) |
| P48066 | SLC6A11 | Sodium- and chloride-dependent GABA transporter 3 | methyl isoheptadecanoate (0.4) |
| O94925 | GLS | Glutaminase kidney isoform, mitochondrial | Macathioamide A (0.47) |
| Q9UBN7 | HDAC6 | Histone deacetylase 6 | Macathioamide A (0.55) |
| P23946 | CMA1 | Chymase | Macathioamide A (0.4) |
| P17252 | PRKCA | Protein kinase C alpha type | erucic acid (0.47), myristoleic acid (0.44) |
| Q13332 | PTPRS | Receptor-type tyrosine-protein phosphatase S | Kaempferol-7-O-alpha-L-rhamnopyranoside (0.44) |
| P30305 | CDC25B | M-phase inducer phosphatase 2 | erucic acid (0.57), myristoleic acid (0.53) |
| P22748 | CA4 | Carbonic anhydrase 4 | Kaempferol-7-O-alpha-L-rhamnopyranoside (0.47), Apetalumoside A (0.66) |
| Q9UBY5 | LPAR3 | Lysophosphatidic acid receptor 3 | myristoleic acid glycine (0.43),9,11,14‑Eicosatrienoic acid glycine (0.4), erucic acid (0.45), myristoleic acid (0.47) |
| Q9NPH5 | NOX4 | NADPH oxidase 4 | Kaempferol-7-O-alpha-L-rhamnopyranoside (0.47) |
| Q9Y253 | POLH | DNA polymerase eta | erucic acid (0.4) |
| Q9P0X4 | CACNA1I | Voltage-dependent T-type calcium channel subunit alpha-1I | Macathioamide A (0.42) |
| K7ENN9 | ALOX12 | Arachidonate 12-lipoxygenase, 12S-type | 9,11,14‑Eicosatrienoic acid glycine (0.6), erucic acid (0.58),3- hydroxy alpha-linolenic acid (0.55), myristoleic acid (0.54) |
| P41252 | IARS1 | Isoleucine--tRNA ligase, cytoplasmic | Glucotropaolin Sulfate (0.31), Benzyl glucosinolate Sulfate (0.3) |
| Q96IY4 | CPB2 | Carboxypeptidase B2 | Cysteine (0.41) |
| P11387 | TOP1 | DNA topoisomerase 1 | erucic acid (0.88), myristoleic acid (0.81) |
| P21397 | MAOA | Amine oxidase [flavin-containing] A | Kaempferol-7-O-alpha-L-rhamnopyranoside (0.48) |
| Q9BXC1 | GPR174 | Probable G-protein coupled receptor 174 | myristoleic acid glycine (0.43),9,11,14‑Eicosatrienoic acid glycine (0.41), erucic acid (0.41) |
| P04035 | HMGCR | 3-hydroxy-3-methylglutaryl-coenzyme A reductase | Benzyl isothiocyanate N5-(3-((benzylcarbamothioyl)thio)-1-((carboxymethyl)amino)-1-oxopropan-2-yl) glutamine (0.4), myristoleic acid glycine (0.45),9,11,14‑Eicosatrienoic acid glycine (0.42), erucic acid (0.65), methyl isoheptadecanoate (0.43), myristoleic acid (0.6) |
| P00915 | CA1 | Carbonic anhydrase 1 | Kaempferol-7-O-alpha-L-rhamnopyranoside (0.62), Macathioamide A (0.44), Apetalumoside A (0.66) |
| P43005 | SLC1A1 | Excitatory amino acid transporter 3 | Cysteine (0.46), Benzyl isothiocyanate N5-(3-((benzylcarbamothioyl)thio)-1-((carboxymethyl)amino)-1-oxopropan-2-yl) glutamine (0.45) |
| O00398 | P2RY10 | Putative P2Y purinoceptor 10 | myristoleic acid glycine (0.43),9,11,14‑Eicosatrienoic acid glycine (0.41), erucic acid (0.41) |
| O15054 | KDM6B | Lysine-specific demethylase 6B | Macathioamide A (0.41) |
| P15121 | AKR1B1 | Aldo-keto reductase family 1 member B1 | Apetalumoside A (0.8), Kaempferol-7-O-alpha-L-rhamnopyranoside (0.62) |
| Q03181 | PPARD | Peroxisome proliferator-activated receptor delta | erucic acid (1), myristoleic acid (0.92) |
| P09923 | ALPI | Intestinal-type alkaline phosphatase | Kaempferol-7-O-alpha-L-rhamnopyranoside (0.46), Apetalumoside A (0.66) |
| Q9P2J5 | LARS1 | Leucine--tRNA ligase, cytoplasmic | Glucotropaolin Sulfate (0.31), Benzyl glucosinolate Sulfate (0.3) |
| P43088 | PTGFR | Prostaglandin F2-alpha receptor | erucic acid (0.41) |
| Q8TDS5 | OXER1 | Oxoeicosanoid receptor 1 | myristoleic acid glycine (0.59),9,11,14‑Eicosatrienoic acid glycine (0.65), erucic acid (0.88),3- hydroxy alpha-linolenic acid (0.69), myristoleic acid (0.81) |

**Table S3: KEGG pathway analysis of potential target genes functions**

| pathway ID | Pathway  description | observed gene count | Enrichment score | P value | matching proteins in network |
| --- | --- | --- | --- | --- | --- |
| hsa05200 | Pathways in cancer | 16 | 0.92 | 3.14E-08 | IL2, PTGER2, FGF2, PPARG, PTGER4, PPARD, GSTM1, PTGER3, LPAR3, MMP9, LPAR1, MAPK8, GSTP1, IL6, PRKCA, HDAC2 |
| hsa01100 | Metabolic pathways | 22 | 0.61 | 1.53E-06 | LTA4H, ALOX12, ALDH2, CA2, AKR1B1, HMGCR, ALPI, ANPEP, CA4, GSTM1, SPHK1, GLS, MAOA, CYP1A2, CYP2C19, XDH, CBS, GSTP1, PLA2G2A, PLA2G10, EPHX2, CA1 |
| hsa04080 | Neuroactive ligand-receptor interaction | 11 | 0.95 | 5.89E-06 | P2RY10, PTGER2, PTGER4, PTGER3, CNR1, LPAR3, PTGFR, LPAR1, CNR2, LTB4R, TRPV1 |
| hsa00590 | Arachidonic acid metabolism | 6 | 1.43 | 1.43E-05 | LTA4H, ALOX12, CYP2C19, PLA2G2A, PLA2G10, EPHX2 |
| hsa04750 | Inflammatory mediator regulation of TRP channels | 6 | 1.24 | 0.00012 | PTGER2, ALOX12, PTGER4, MAPK8, PRKCA, TRPV1 |
| hsa05206 | MicroRNAs in cancer | 7 | 1.07 | 0.00016 | CDC25B, GLS, MMP9, ABCC1, PRKCA, HDAC2, ABCB1 |
| hsa00591 | Linoleic acid metabolism | 4 | 1.56 | 0.00029 | CYP1A2, CYP2C19, PLA2G2A, PLA2G10 |
| hsa00982 | Drug metabolism - cytochrome P450 | 5 | 1.32 | 0.00029 | GSTM1, MAOA, CYP1A2, CYP2C19, GSTP1 |
| hsa01523 | Antifolate resistance | 4 | 1.54 | 0.00032 | ALOX12, ABCC1, IL6, ABCG2 |
| hsa04972 | Pancreatic secretion | 5 | 1.14 | 0.0014 | CPB2, CA2, PLA2G2A, PLA2G10, PRKCA |
| hsa00910 | Nitrogen metabolism | 3 | 1.67 | 0.0017 | CA2, CA4, CA1 |
| hsa04964 | Proximal tubule bicarbonate reclamation | 3 | 1.56 | 0.0031 | CA2, CA4, GLS |
| hsa00983 | Drug metabolism - other enzymes | 4 | 1.16 | 0.0054 | MPO, GSTM1, XDH, GSTP1 |
| hsa05204 | Chemical carcinogenesis | 4 | 1.15 | 0.0055 | GSTM1, CYP1A2, CYP2C19, GSTP1 |
| hsa04072 | Phospholipase D signaling pathway | 5 | 0.96 | 0.0059 | SPHK1, LPAR3, PTGFR, LPAR1, PRKCA |
| hsa00232 | Caffeine metabolism | 2 | 2.03 | 0.006 | CYP1A2, XDH |
| hsa04151 | PI3K-Akt signaling pathway | 7 | 0.73 | 0.0072 | IL2, TLR2, FGF2, LPAR3, LPAR1, IL6, PRKCA |
| hsa04976 | Bile secretion | 4 | 1.08 | 0.0072 | CA2, HMGCR, ABCB1, ABCG2 |
| hsa05161 | Hepatitis B | 5 | 0.93 | 0.0072 | TLR2, MMP9, MAPK8, IL6, PRKCA |
| hsa04657 | IL-17 signaling pathway | 4 | 1.07 | 0.0075 | MMP9, MAPK8, ELAVL1, IL6 |
| hsa04933 | AGE-RAGE signaling pathway in diabetic complications | 4 | 1.05 | 0.0083 | NOX4, MAPK8, IL6, PRKCA |
| hsa05142 | Chagas disease | 4 | 1.04 | 0.0083 | IL2, TLR2, MAPK8, IL6 |
| hsa05202 | Transcriptional misregulation in cancer | 5 | 0.89 | 0.0083 | MPO, PPARG, MMP9, IL6, HDAC2 |
| hsa00380 | Tryptophan metabolism | 3 | 1.29 | 0.0084 | ALDH2, MAOA, CYP1A2 |
| hsa02010 | ABC transporters | 3 | 1.25 | 0.0104 | ABCC1, ABCB1, ABCG2 |
| hsa04010 | MAPK signaling pathway | 6 | 0.75 | 0.0104 | CDC25B, FGF2, RPS6KA3, MAPK8, CACNA1I, PRKCA |
| hsa04020 | Calcium signaling pathway | 5 | 0.84 | 0.0104 | SPHK1, PTGER3, PTGFR, CACNA1I, PRKCA |
| hsa04726 | Serotonergic synapse | 4 | 0.99 | 0.0104 | ALOX12, MAOA, CYP2C19, PRKCA |
| hsa04931 | Insulin resistance | 4 | 1 | 0.0104 | RPS6KA3, MAPK8, PPARA, IL6 |
| hsa05144 | Malaria | 3 | 1.24 | 0.0104 | TLR2, SELP, IL6 |
| hsa04015 | Rap1 signaling pathway | 5 | 0.82 | 0.0114 | FGF2, CNR1, LPAR3, LPAR1, PRKCA |
| hsa04071 | Sphingolipid signaling pathway | 4 | 0.96 | 0.0114 | SPHK1, MAPK8, ABCC1, PRKCA |
| hsa00480 | Glutathione metabolism | 3 | 1.19 | 0.0117 | ANPEP, GSTM1, GSTP1 |
| hsa05135 | Yersinia infection | 4 | 0.93 | 0.0134 | IL2, RPS6KA3, MAPK8, IL6 |
| hsa05163 | Human cytomegalovirus infection | 5 | 0.79 | 0.0141 | PTGER2, PTGER4, PTGER3, IL6, PRKCA |
| hsa05418 | Fluid shear stress and atherosclerosis | 4 | 0.92 | 0.0146 | GSTM1, MMP9, MAPK8, GSTP1 |
| hsa05321 | Inflammatory bowel disease | 3 | 1.13 | 0.0147 | IL2, TLR2, IL6 |
| hsa04014 | Ras signaling pathway | 5 | 0.77 | 0.0151 | FGF2, MAPK8, PLA2G2A, PLA2G10, PRKCA |
| hsa05162 | Measles | 4 | 0.89 | 0.0167 | IL2, TLR2, MAPK8, IL6 |
| hsa05031 | Amphetamine addiction | 3 | 1.09 | 0.0178 | MAOA, PRKCA, HDAC2 |
| hsa04723 | Retrograde endocannabinoid signaling | 4 | 0.87 | 0.018 | FAAH, CNR1, MAPK8, PRKCA |
| hsa00980 | Metabolism of xenobiotics by cytochrome P450 | 3 | 1.06 | 0.0199 | GSTM1, CYP1A2, GSTP1 |
| hsa01524 | Platinum drug resistance | 3 | 1.06 | 0.0203 | GSTM1, POLH, GSTP1 |
| hsa03320 | PPAR signaling pathway | 3 | 1.03 | 0.0239 | PPARG, PPARD, PPARA |
| hsa00340 | Histidine metabolism | 2 | 1.4 | 0.0248 | ALDH2, MAOA |
| hsa01521 | EGFR tyrosine kinase inhibitor resistance | 3 | 1.02 | 0.0248 | FGF2, IL6, PRKCA |
| hsa05152 | Tuberculosis | 4 | 0.81 | 0.0266 | TLR2, SPHK1, MAPK8, IL6 |
| hsa04614 | Renin-angiotensin system | 2 | 1.36 | 0.0274 | CMA1, ANPEP |
| hsa04727 | GABAergic synapse | 3 | 0.97 | 0.0301 | SLC6A11, GLS, PRKCA |
| hsa00592 | alpha-Linolenic acid metabolism | 2 | 1.33 | 0.0307 | PLA2G2A, PLA2G10 |
| hsa00790 | Folate biosynthesis | 2 | 1.33 | 0.0307 | AKR1B1, ALPI |
| hsa04914 | Progesterone-mediated oocyte maturation | 3 | 0.92 | 0.0383 | CDC25B, RPS6KA3, MAPK8 |
| hsa05169 | Epstein-Barr virus infection | 4 | 0.74 | 0.0398 | TLR2, MAPK8, IL6, HDAC2 |
| hsa04659 | Th17 cell differentiation | 3 | 0.91 | 0.0405 | IL2, MAPK8, IL6 |
| hsa05205 | Proteoglycans in cancer | 4 | 0.74 | 0.0405 | TLR2, FGF2, MMP9, PRKCA |
| hsa04620 | Toll-like receptor signaling pathway | 3 | 0.9 | 0.0409 | TLR2, MAPK8, IL6 |
| hsa04625 | C-type lectin receptor signaling pathway | 3 | 0.9 | 0.0412 | IL2, MAPK8, IL6 |
| hsa05146 | Amoebiasis | 3 | 0.9 | 0.0412 | TLR2, IL6, PRKCA |
| hsa04024 | cAMP signaling pathway | 4 | 0.71 | 0.0461 | PTGER2, PTGER3, MAPK8, PPARA |
| hsa04668 | TNF signaling pathway | 3 | 0.86 | 0.0499 | MMP9, MAPK8, IL6 |
| hsa04724 | Glutamatergic synapse | 3 | 0.85 | 0.0499 | SLC1A1, GLS, PRKCA |
| hsa05143 | African trypanosomiasis | 2 | 1.17 | 0.0499 | IL6, PRKCA |
| hsa05332 | Graft-versus-host disease | 2 | 1.17 | 0.0499 | IL2, IL6 |

**
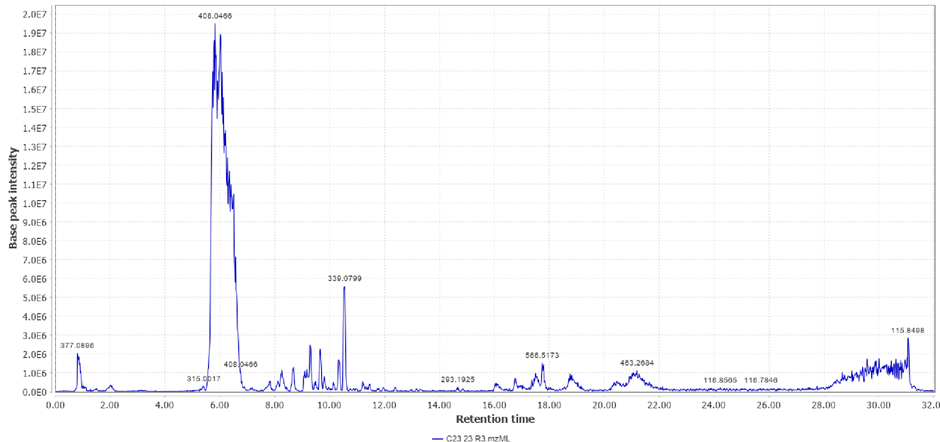
**

A

**
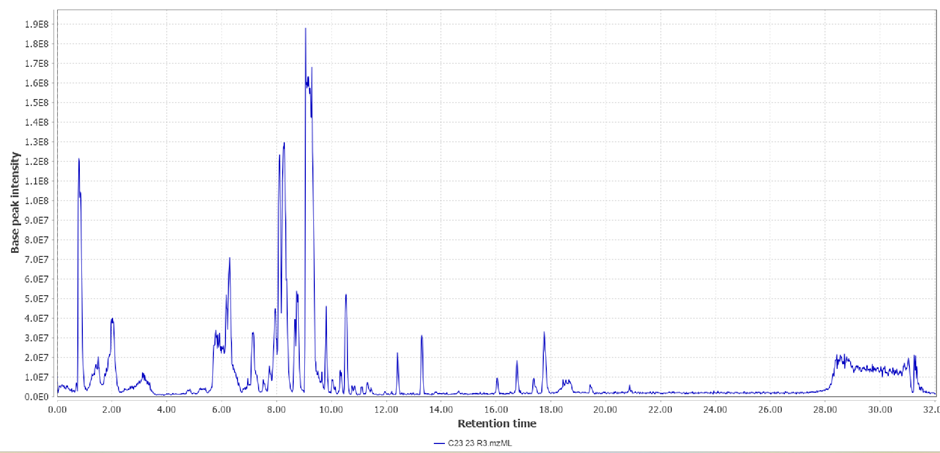
**

B

**Figure S1: UPLC-MS/MS base peak chromatograms of *L. sativum* seeds ethanolic extract. In negative ionization mode (A) and in positive ionization mode (B).**

**
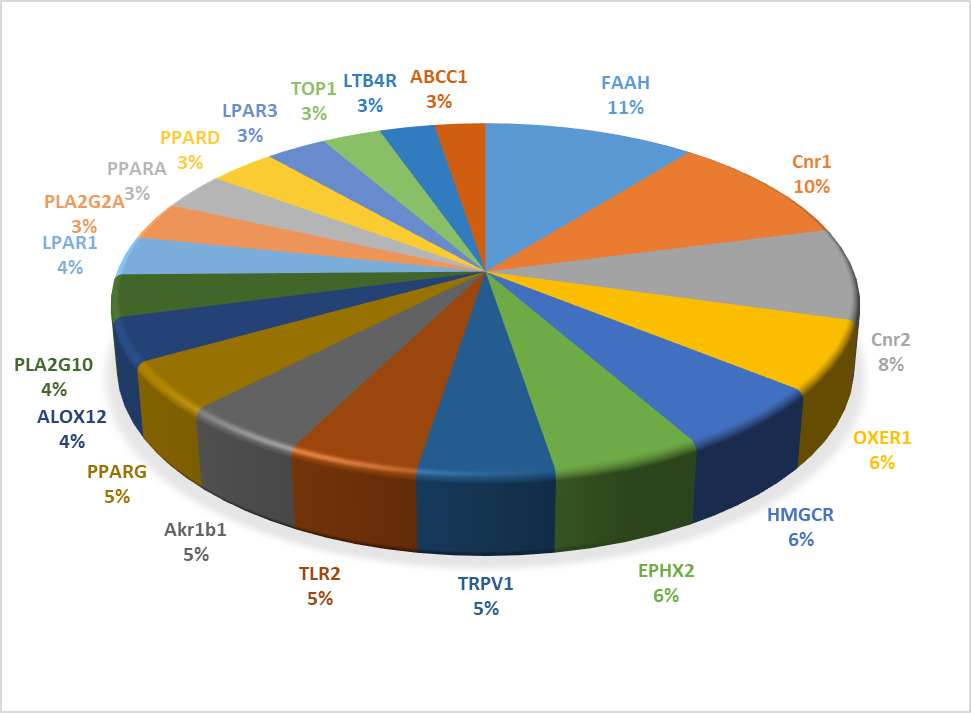
**

**Figure S2: The** **distribution percentage of top 20 potential targets affected by *L. sativum* absorbed serum metabolites.**

**Figure S3: The distribution percentage of serum metabolites after administration of the ethanolic extract of *L. sativum seeds*.**

**References**

1. Le A, Ng A, Kwan T, Cusmano-Ozog K, Cowan TM. A rapid, sensitive method for quantitative analysis of underivatized amino acids by liquid chromatography–tandem mass spectrometry (LC–MS/MS). J Chromatogr B [Internet]. 2014 Jan;944:166–74. Available from: https://linkinghub.elsevier.com/retrieve/pii/S1570023213006302

2. Ibrahim RM, Elmasry GF, Refaey RH, El-Shiekh RA. Lepidium meyenii (Maca) Roots: UPLC-HRMS, Molecular Docking, and Molecular Dynamics. ACS Omega. 2022;7(20):17339–57.

3. Liu M, Zhang D, Wang X, Zhang L, Han J, Yang M, et al. Simultaneous quantification of niacin and its three main metabolites in human plasma by LC-MS/MS. Vol. 904, Journal of Chromatography B: Analytical Technologies in the Biomedical and Life Sciences. 2012. p. 107–14.

4. Abdallah HM, Farag MA, Algandaby MM, Nasrullah MZ, Abdel-Naim AB, Eid BG, et al. Osteoprotective activity and metabolite fingerprint via uplc/ms and gc/ms of lepidium sativum in ovariectomized rats. Vol. 12, Nutrients. 2020. p. 1–20.

5. Ibrahim RS, El-Mezayen NS, El‐Banna AA. Alleviation of liver cirrhosis and associated portal-hypertension by Astragalus species in relation to their UPLC-MS/MS metabolic profiles: a mechanistic study. Sci Rep [Internet]. 2022;12(1):1–27. Available from: https://doi.org/10.1038/s41598-022-15958-1

6. Hamed AI, Said R Ben, Kontek B, Al-Ayed AS, Kowalczyk M, Moldoch J, et al. LC–ESI-MS/MS profile of phenolic and glucosinolate compounds in samh flour ( Mesembryanthemum forsskalei Hochst. ex Boiss) and the inhibition of oxidative stress by these compounds in human plasma. Food Res Int [Internet]. 2016 Jul;85:282–90. Available from: http://dx.doi.org/10.1016/j.foodres.2016.04.009

7. Tafuri S, Cocchia N, Carotenuto D, Vassetti A, Staropoli A, Mastellone V, et al. Chemical analysis of Lepidium meyenii (Maca) and its effects on redox status and on reproductive biology in stallions. Molecules. 2019;24(10):1–12.

8. Wang S, Li X, Chen B, Li S, Wang J, Wang J, et al. Dimension-enhanced ultra-high performance liquid chromatography/ion mobility-quadrupole time-of-flight mass spectrometry combined with intelligent peak annotation for the rapid characterization of the multiple components from seeds of descurainia sophia. Phyton-International J Exp Bot. 2022;91(3):541–67.

9. Anders Kjaer, Mamoru Ohashi , J. Michael Wilson CD and JMP. Mass spectra of isothiocyanates. Acta Chem Scand [Internet]. 1963;17:2143–54. Available from: https://api.semanticscholar.org/CorpusID:95059588

10. Nicolescu TO. Interpretation of Mass Spectra. Mass Spectrom [Internet]. 2017; Available from: http://dx.doi.org/10.5772/intechopen.68595

11. Rodig OR. Spectrometric Identification of Organic Compounds. J Med Chem [Internet]. 1963 Nov 1;6(6):826–7. Available from: https://pubs.acs.org/doi/abs/10.1021/jm00342a059

12. SUZUKI T, TUZIMURA K. Mass spectra of amino acid hydantoins. Agric Biol Chem [Internet]. 1976;40(1):225–6. Available from: http://www.jstage.jst.go.jp/article/bbb1961/40/1/40_1_225/_article

13. Bennouna D, Avice JC, Rosique C, Svilar L, Pontet C, Trouverie J, et al. The impact of genetics and environment on the polar fraction metabolome of commercial Brassica napus seeds: a multi-site study. Seed Sci Res [Internet]. 2019 Sep 5;29(3):167–78. Available from: https://www.cambridge.org/core/product/identifier/S0960258519000138/type/journal_article

14. Ousji O, Sleno L. Structural Elucidation of Novel Stable and Reactive Metabolites of Green Tea Catechins and Alkyl Gallates by LC-MS/MS. Antioxidants [Internet]. 2022 Aug 23;11(9):1635. Available from: https://www.mdpi.com/2076-3921/11/9/1635

15. Oszmiański J, Kolniak-Ostek J, Wojdyło A. Application of ultra performance liquid chromatography-photodiode detector-quadrupole/time of flight-mass spectrometry (UPLC-PDA-Q/TOF-MS) method for the characterization of phenolic compounds of Lepidium sativum L. sprouts. Eur Food Res Technol. 2013;236(4):699–706.

16. Maldini M, Foddai M, Natella F, Petretto GL, Rourke JP, Chessa M, et al. Identification and quantification of glucosinolates in different tissues of Raphanus raphanistrum by liquid chromatography tandem-mass spectrometry. J Food Compos Anal [Internet]. 2017;61:20–7. Available from: http://dx.doi.org/10.1016/j.jfca.2016.06.002

17. Della Corte A, Chitarrini G, Di Gangi IM, Masuero D, Soini E, Mattivi F, et al. A rapid LC-MS/MS method for quantitative profiling of fatty acids, sterols, glycerolipids, glycerophospholipids and sphingolipids in grapes. Talanta [Internet]. 2015;140:52–61. Available from: http://dx.doi.org/10.1016/j.talanta.2015.03.003
